# Plant-based nanoparticles targeting malaria management

**DOI:** 10.3389/fphar.2024.1440116

**Published:** 2024-08-09

**Authors:** Pathy B. Lokole, Galilée G. Byamungu, Paulin K. Mutwale, Nadège K. Ngombe, Celestin N. Mudogo, Rui W. M. Krause, Christian I. Nkanga

**Affiliations:** ^1^ Centre de Recherche en Nanotechnologies Appliquées aux Produits Naturels (CReNAPN), Department of Medicinal Chemistry and Pharmacognosy, Faculty of Pharmaceutical Sciences, University of Kinshasa, Kinshasa, Democratic Republic of the Congo; ^2^ Centre d’Etudes des Substances Naturelles d’Origine Végétale (CESNOV), Faculty of Pharmaceutical Sciences, University of Kinshasa, Kinshasa, Democratic Republic of the Congo; ^3^ Center for Chemico- and Bio-Medicinal Research (CCBR), Department of Chemistry, Faculty of Sciences, Rhodes University, Grahamstown, Eastern Cape, South Africa; ^4^ Department of Chemistry, Faculty of Sciences and Technology, University of Kinshasa, Kinshasa, Democratic Republic of the Congo; ^5^ Unit of Molecular Biology, Department of Basic Sciences, Faculty of Medicine, University of Kinshasa, Kinshasa, Democratic Republic of the Congo

**Keywords:** medicinal plants, antimalarial drugs, insecticides, green synthesis, nanoparticles, targeted drug delivery, Plasmodium spp

## Abstract

Malaria is one of the most devastating diseases across the globe, particularly in low-income countries in Sub-Saharan Africa. The increasing incidence of malaria morbidity is mainly due to the shortcomings of preventative measures such as the lack of vaccines and inappropriate control over the parasite vector. Additionally, high mortality rates arise from therapeutic failures due to poor patient adherence and drug resistance development. Although the causative pathogen (*Plasmodium* spp.) is an intracellular parasite, the recommended antimalarial drugs show large volumes of distribution and low-to no-specificity towards the host cell. This leads to severe side effects that hamper patient compliance and promote the emergence of drug-resistant strains. Recent research efforts are promising to enable the discovery of new antimalarial agents; however, the lack of efficient means to achieve targeted delivery remains a concern, given the risk of further resistance development. New strategies based on green nanotechnologies are a promising avenue for malaria management due to their potential to eliminate malaria vectors (Anopheles sp.) and to encapsulate existing and emerging antimalarial agents and deliver them to different target sites. In this review we summarized studies on the use of plant-derived nanoparticles as cost-effective preventative measures against malaria parasites, starting from the vector stage. We also reviewed plant-based nanoengineering strategies to target malaria parasites, and further discussed the site-specific delivery of natural products using ligand-decorated nanoparticles that act through receptors on the host cells or malaria parasites. The exploration of traditionally established plant medicines, surface-engineered nanoparticles and the molecular targets of parasite/host cells may provide valuable insights for future discovery of antimalarial drugs and open new avenues for advancing science toward the goal of malaria eradication.

## 1 Introduction

Malaria is one of the deadliest and most widespread infectious diseases worldwide. The 2022 report of the World Health Organization (WHO) revealed that in 2021, malaria caused 619,000 deaths and 247 million infections globally (WHO, 2022).

Middle- and low-income countries remain the most affected by the malaria epidemic, largely due to their weaker and less comprehensive strategic approaches. Rather than decreasing, the prevalence of malaria is increasing annually, particularly among the most vulnerable groups such as children under 5 years and pregnant women. Malaria infection during pregnancy poses a high risk of morbidity and mortality for both the mother and infant (Bauserman et al., 2019; Dosoo et al., 2020).

Studies indicate that malaria also significantly weakens the economies of affected countries, especially those classified as with “medium and low income”, not least because measures for its eradication are costly (Sarma et al., 2019). In mitigation, financial and logistical aid organizations are providing substantial support to these countries to help them combat malaria.

Considerable efforts have been made since the last century to eradicate malaria globally. Eradication strategies primarily focus on vector control, which target the Anopheles mosquitoes (Sato, 2021) in both larval and adult stages, particularly in stagnant waters and wetlands. Commonly used agents include dichlorodiphenyltrichloroethane (DDT), synthetic pyrethroids, and organophosphates, which are utilized in indoor and outdoor residual spraying against Anopheles mosquitoes (Raghavendra et al., 2011). Given the toxicity of these traditional chemical agents and their long-term effects on human health and the environment, there is a growing focus on natural products, especially essential oils (EOs), which are monoterpenoid compounds from plant extracts (He et al., 2018; Asadollahi et al., 2019; Sheikh et al., 2021a). These natural alternatives are garnering attention due to their lower toxicity, availability worldwide, and affordability. They are being explored for their efficacy in repelling and eliminating female Anopheles (Moemenbellah-Fard et al., 2021). Although EOs are eco-friendly products and have shown potential insecticide and repellent activities, some remain less effective than synthetic pyrethroids, especially those with low monoterpenoid concentrations (Norris et al., 2015). Previous studies showed that adding vanillin 5% (as a fixative compound) to essential oils can improve their duration as a repellent against mosquito bites (Auysawasdi et al., 2016). Additionally, antivectorial control strategies are evolving to include the spraying of mosquito repellents in homes and the distributing of insecticide-impregnated mosquito nets to at-risk populations (Dosoo et al., 2020).

Malaria’s persistence can also be attributed to multiple policy factors, including the lack- or inadequate-implementation of environmental sanitation policies. Such policy gaps contribute to the prevalence of stagnant water in urban areas, which serve as breeding grounds for mosquitoes (Sinden, 2015; Subramaniam et al., 2016). This leads to the continued survival and proliferation of the mosquito populations responsible for spreading malaria (Meibalan and Marti, 2017). Several pesticides possessing larvicidal properties are employed to eliminate mosquito larvae in marshlands. However, these chemicals pose significant toxicity risks to aquatic environments and their entire biomass (Mohammadi et al., 2021). Additionally, resistance to these larvicides has been observed in several regions across the globe (Raghavendra et al., 2011). Another critical issue is the developing resistance of female Anopheles mosquitoes to insecticides used in insecticide-impregnated mosquito nets (Ngufor et al., 2016). In middle- and low-income countries, limited access to healthcare facilities and qualified medical personnel often results in delayed malaria diagnosis and the commencement of appropriate treatment. This delay not only exacerbates the infection but can also, tragically, lead to patient fatalities (World Health Organization, 2022). The combination of growing insecticide resistance and healthcare accessibility challenges underscores the need for a multifaceted approach to malaria control and treatment.

In 2012, the WHO set up the global plan for insecticide resistance management in malaria (GPIRM), which comprises five strategies to be implemented in countries affected by insecticide-resistant mosquitoes, and which form the pillars of the fight against insecticide resistance: i) plan and implement insecticide resistance management strategies in malaria-endemic countries; ii) ensure proper, timely entomological and resistance monitoring and effective data management; iii) develop new, innovative vector control tools; iv) fill gaps in knowledge on mechanisms of insecticide resistance and the impact of current insecticide resistance management strategies; and v) ensure that the appropriate advocacy, human and financial resources are in place (World Health Organization, 2012; Mnzava et al., 2015).

**FIGURE 1:**
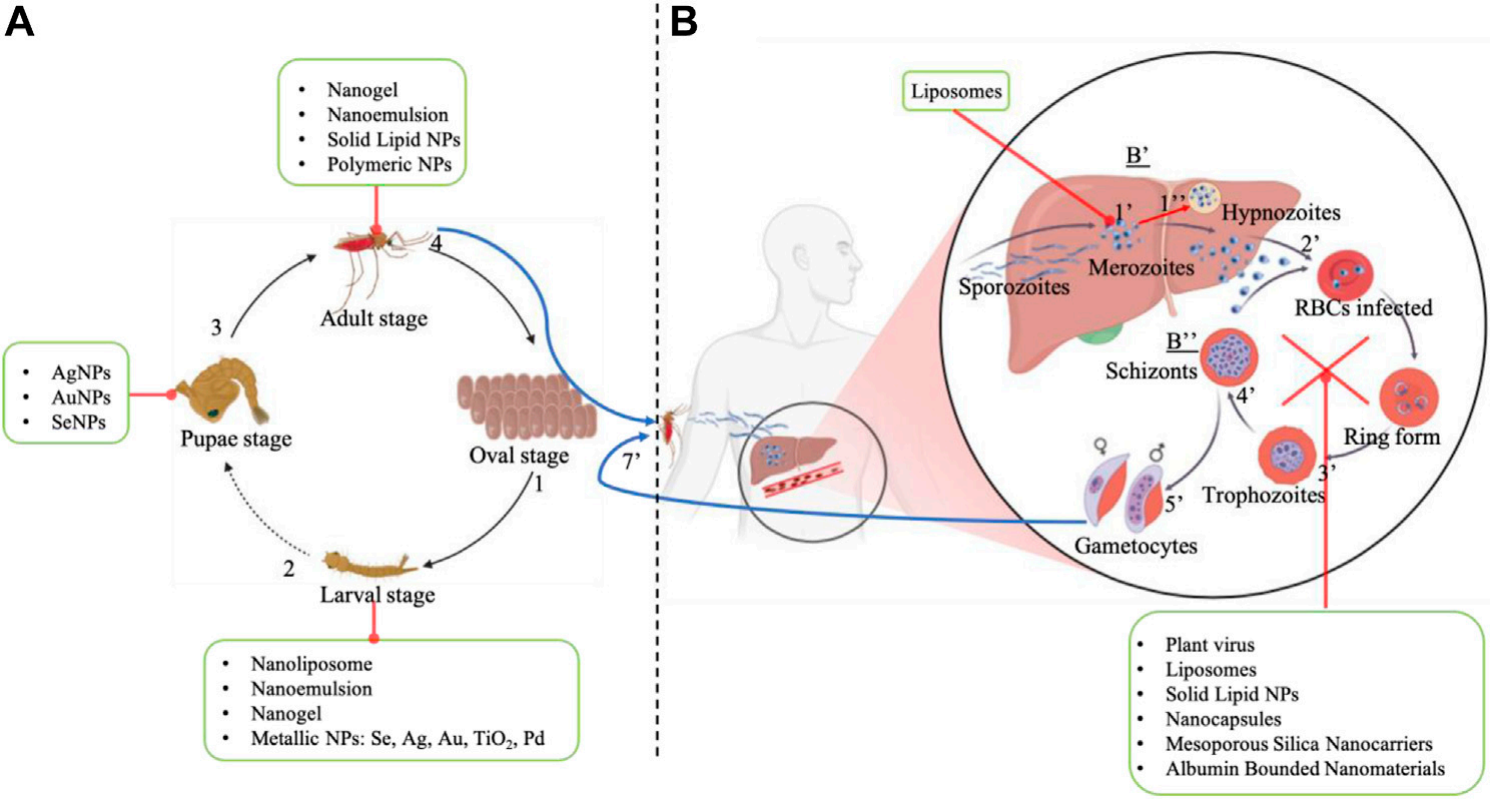
Schematic illustration of the Life Cycle of anopheles mosquito **(A)** and Transmission cycle of plasmodium parasites **(B)**. The life cycle of the Anopheles mosquito is divided into 4 stages: the female lays eggs in swampy areas (1), these eggs develop into larvae (2) within a few days, these larvae grow and mutate into pupae (3), which mature into adult mosquitoes (4). To ensure its survival, the hematophagous Anopheles feeds on human blood, and it’s during this blood meal that the Anopheles, a vector of Plasmodium, infects humans and conducts to the transmission cycle of plasmodium in human beings. Plasmodium parasites in humans develop in two phases: hepatocytes (B’) and erythrocytes (B″) stages. In the first stage (B’), the parasite undergoes perpetual mutation from sporozoites to merozoites (1’) in liver cells, and particularly for *P. ovale* and *P. vivax*, merozoites can enter in a period of snooze, forming hypnozoites (1’’). This phase is usually asymptomatic and can last from a few hours to a few days for *P. falciparum* and several days for *P. ovale* and *P. vivax*. When sufficiently colonized, hepatocytes are lyzed and merozoites are released into the bloodstream, then start infection of erythrocytes (B″). This phase begins with merozoites invading RBCs (2’’) and transforming into trophozoites (3’’) then into schizonts (4’’), which infect other RBCs. At the end of this phase, trophozoites differentiate into male and female gametocytes (5’’), which are consumed by non-infected mosquitoes (7’’) The gametocytes that infect the female mosquitoes undergo several mutations in the digestive tract, before finally transforming into sporozoites that migrate to the mosquito’s salivary glands and are injected into man during the mosquito’s next blood meal. In this way, malaria spreads from person to person via the various mutations in humans and Anopheles mosquitoes. Illustration conceived with www.BioRender.com.

Nanotechnologies are being used to develop innovative tools for controlling the vectors of parasitic diseases, particularly malaria (Figure 1). Research has shown that metallic nanoparticles synthesized from plants and plant extracts are effective against mosquito eggs, larvae, pupae, and adults, and can target Anopheles mosquitoes (Onen et al., 2023). These bio-derived nanoparticles are referred to as “Green nanoparticles” due to the use of low-toxicity reagents in their synthesis, and may have many advantages over traditional insecticides, including that they are easy to formulate, have low toxicity and have no known resistance to date (Auysawasdi et al., 2016).

The fight against malaria also involves downstream efforts, such as intermittent preventive treatments to protect at-risk populations from plasmodium parasite infection. Beyond any preventive tools, vaccines remain the most efficient tools for protecting humans against malaria (Laurens, 2019). This is why the WHO recommends systemic integration of vaccination policies in malaria-impacted countries.

In 2019, WHO endorsed using the RTS, S/AS01 vaccine Mosquirix^®^ by GlaxoSmithKline (GSK) in Ghana, Kenya, and Malawi (World Health Organization, 2023).

By 2023, the rollout of the RTS,S/AS01 vaccine allowed the vaccination of more than 2 million children in these countries, resulting in a decrease in childhood deaths by 13% (World Health Organization, 2023). The same WHO report highlighted that a second vaccine named R21/Matrix-MTM (Oxford University and Serum Institute of India) was recommended in October 2023, and both vaccines are safe and effective in reducing malaria disease and burden cases.

In recent years, several African countries have reached out to the WHO and its partners, seeking to integrate malaria vaccines into their public health strategies. While the deployment of these vaccines marks a significant milestone, the fight against malaria is far from over, with several other vaccine candidates continuing to undergo development and testing. Among these candidates, many formulations have reached clinical trials, one of which is PfSPZ-Vac (*P. falciparum* sporozoite chemoprophylaxis vaccine) developed by Sanaria Inc. (Stanisic and Good, 2023). Critically, however, while the vaccine rollout continues, the WHO advocates for the provision of effective and affordable anti-malaria medications to all patients suffering from malaria (Laurens, 2019).

The most frequently-used antimalarial agents include analogues or derivatives of existing drugs such as quinine and artemisinin. These were initially sourced from the plant kingdom, with quinine isolated from *Cinchona officinalis* and artemisinin from *Artemisia annua* (Misra et al., 2008; Kapepula et al., 2020). Beyond these medicinal plants, numerous other plant species have shown substantial antiplasmodial properties. This efficacy is attributed to their rich composition of bioactive secondary metabolites, which are effective against both malaria vectors (larvae or adult mosquitoes) for the prevention and treatment of malaria. Medicinal plants are increasingly valued as a source of antimalarial agents, primarily due to their affordability, and accessibility (Mukubwa et al., 2020; Moemenbellah-Fard et al., 2021; Ojemaye et al., 2021). Consequently, they represent a viable and effective option for low-income populations to treat not only malaria but also various other diseases.

The eradication of malaria faces challenges due to the pharmacokinetic limitations of antimalarial drugs. Given that plasmodium is an intracellular parasite, it necessitates the use of highly specific drugs and drug delivery for effective treatment (Yaméogo et al., 2020). Antimalarial drugs such as artemisinin derivatives exhibit low oral bioavailability, for example. This issue requires the administration of increased dosages to achieve the necessary concentration within erythrocytes, which can lead to more adverse effects and higher toxicity. Drug resistance renders currently available medicines less effective, again requiring higher therapeutic doses (Haldar et al., 2018; Ferreira et al., 2021; Rasmussen et al., 2022).

To address these challenges and maintain the efficacy of antimalarials for as long as possible, several strategies have been employed. These include the development of rapid malaria diagnostic tests to facilitate early and accurate diagnosis, even of drug-resistance (Wilson, 2013; Sinha et al., 2015; Mbanefo and Kumar, 2020), *in vivo* assessments to evaluate the effectiveness of antimalarial drugs, and *in vitro*/*ex vivo* tests to determine the susceptibility of Plasmodium strains to antimalarial drugs (Nsanzabana et al., 2018). Additionally, ensuring the availability of low-cost, high-quality antimalarial drugs, often supported by donor funding, is a critical component of these efforts (WHO, 2022).

Within the array of strategies for malaria eradication, nanotechnology has arisen as a pivotal approach in the development of effective antimalarial drugs, mainly through the advancement of drug delivery systems (Singh, 2012; Kirtane et al., 2021; Memvanga and Nkanga, 2021). Nanotechnology involves manipulating atomic, molecular and supramolecular materials to create nanoscale particles, known as “nanoparticles” (NPs). This category includes liposomes, micelles and nanoemulsions, (Thakkar and Brijesh, 2016), all of which are employed as drug delivery systems (DDS). Through functionalization at the molecular level, NPs have the potential to enhance the effectiveness of conventional drugs significantly (Nkanga and Krause, 2019). Given their encapsulation or loading potential, NPs address critical issues of traditional drugs, such as low solubility, poor bioavailability and absorption in the body, and limited specificity in targeting receptors found on larvae, parasites and the host, thereby offering a more efficient and targeted approach in malaria treatment (Mohammadi et al., 2021).

Numerous studies have shown that nanotechnologies enhance the pharmacokinetic profiles of existing antimalarials (Santos-Magalhães and Mosqueira, 2010; Chaves et al., 2022) as well as the larvicidal potential of plant-based metallic nanoparticles (Neves Borgheti-Cardoso et al., 2020; Ferreira et al., 2021; Rasmussen et al., 2022). The primary goals of nanotechnology investigations include improving the water solubility and oral bioavailability of active pharmaceutical ingredients (API), reducing their toxicity through encapsulation in nanocarriers, utilizing them in the reduction of metal ions to create metallic nanoparticles with inherent antivectorial or antiplasmodial activity, and employing them as raw materials in the formulation of drug delivery systems (DDSs) (Govindarajan and Benelli, 2016b; Mohammadi et al., 2021; Yadav et al., 2023). Additionally, efforts are made to functionalize nanocarriers to specifically target malaria-related sites, enhancing the precision and efficacy of treatments.

This paper provides a comprehensive review of plant materials used in two keyways: as cost-effective sources for creating intrinsically active nanoparticles and as bases for encapsulating both existing and emerging antimalarial agents, including plant extracts, fractions and pure compounds. This strategy targets both the vector and human stages of *Plasmodium falciparum*, aiming for the comprehensive eradication of malaria. The review highlights the strategic use of plant-based nanotechnologies in combating malaria, addressing multiple lifecycle stages of the parasite. It details the molecular targets of malaria, with a particular focus on the surface functionalization of nanoparticles, a critical technique for enhancing the targeted delivery and efficacy of plant-based antimalarial drugs in both prevention and treatment.

## 2 Plant-based nanoparticles and molecular targets of malaria chemotherapy

Plant-based nanoparticles have emerged as a promising tool in the fight against malaria, with studies showing their effectiveness as ovicides, larvicides, pupicides, adulticides and oviposition deterrents against mosquito vectors (Benelli, 2015; 2016; Mohammadi et al., 2021). These nanoparticles, particularly those synthesized using phytochemicals, are highly toxic against the *An. stephensi* mosquito, a key Malaria vector (Marimuthu et al., 2011; Benelli, 2016). Lipid nanocarriers have been explored for their potential in Malaria chemotherapy, offering a platform to formulate antimalarial drugs and modify their pharmacokinetic profile (Jain et al., 2014). The use of nanotechnology-based carriers, including liposomes and polymeric nanovesicles, has also been investigated for the effective delivery of antimalarials (Rashidzadeh et al., 2021). These carriers have shown promise in minimizing the side effects of drug therapy and enhancing the targeting of antimalarials to infected cells (Santos-Magalhães and Mosqueira, 2010). Furthermore, the identification of new molecular targets within the malaria parasite, such as histone deacetylase and aminopeptidases, has opened up possibilities for the development of new antimalarial agents (Gardiner et al., 2009; Van der Watt et al., 2022).

The use of gold nanoparticles as nano vaccines for Malaria, particularly those targeting the *P. falciparum* antigen Pfs25, has also been explored (Kumar et al., 2015). The use of green synthesized nanoparticles, particularly those with antiplasmodial activity, has also been explored in the fight against Malaria (Murugan et al., 2015).

Surface-engineered nanoparticles have shown promise in targeting and inhibiting *P. falciparum*. Varela-Aramburu et al. (2020) developed glucose-based ultra-small gold nanoparticles or gold nanoclusters (Glc-NCs) that bind to cysteine-rich domains of *P. falciparum* surface proteins, enhancing the delivery of antimalarial drugs. *P. falciparum* surface proteins contain cysteine-rich domains that play important roles during the *P. falciparum* invasion process, such as the reticulocyte binding homolog 5 (PfRh5), the cysteine-rich protective antigen (CyRPA), the erythrocyte-binding antigen-175 (EBA-175), the cysteine repeat molecular proteins (PfPCRMP1-2), or the Duffy-binding like erythrocyte-binding protein (DBL-EBP); cysteine-rich domains are expressed on the surface of schizonts, gametocytes and sporozoites of *P. falciparum* (Gardiner et al., 2009; Kumar et al., 2015; Rashidzadeh et al., 2021).

## 3 Nanoengineering strategies against malaria disease

The application of nanoparticles in malaria management encompasses both preventive and curative pathways (Figure 1). The preventive pathway focuses on mosquito control engineering, which includes the use of mosquito repellents and larvicidal platforms. The curative path involves nanoparticle-mediated administration of antimalarial drugs for treating the disease. This dual approach leverages the unique properties of nanoparticles to enhance mosquito control measures and improve the efficacy of antimalarial drugs (Dubey and Mostafavi, 2023; Kekani and Witika, 2023).

**FIGURE 2:**
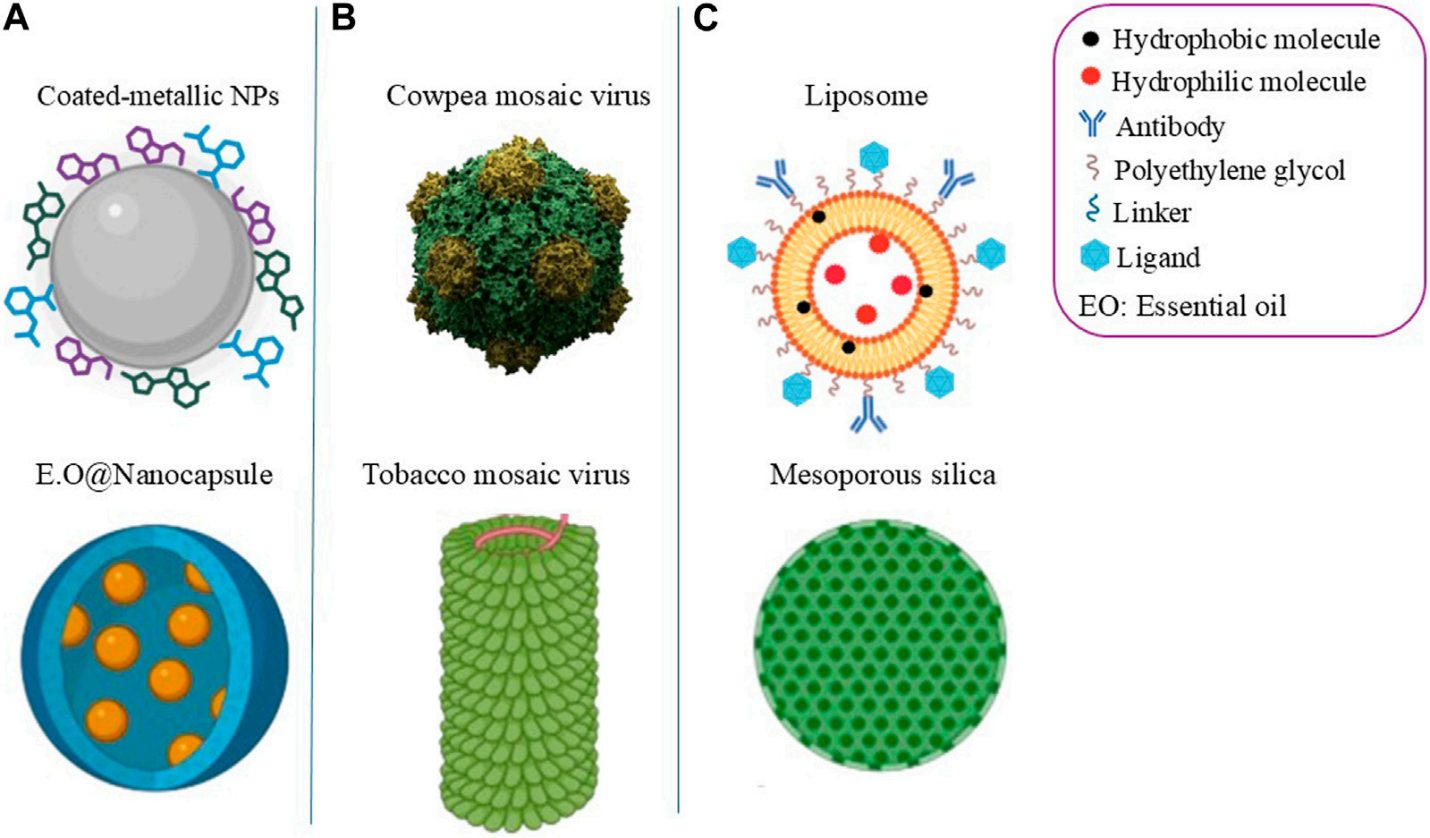
Illustrative structures of plant-based nanoparticles used to manage malaria. **(A)** Top: Plant extract-based metallic NPs coated with phytochemicals. Bottom: essential oil encapsulated in nanocapsule, both for the vector control. **(B)** Plant virus-based vaccine and antimalarial nanocarriers from mosaic viruses. **(C)** Nanocarriers encapsulating existing antimalarials or plant extract with antimalarial potential. Structures created with www.BioRender.com.

Various forms of nanomaterials, including liposomes, nanoemulsions, polymeric, and inorganic nanoparticles (as illustrated in Figure 2) have been developed for the management of malaria. Although the use of nanoparticles seems to be the ideal solution to solve the malaria problem, it has been reported that chemically synthesized nanoparticles exhibit high toxicity on eukaryotic cells due to the presence of synthetic chemicals as capping agents and surface functional handles (Egbuna et al., 2021; Anand et al., 2022). Recently, there has been a growing interest in the formulation of plant-based nanoparticles, particularly due to their biocompatibility, affordability, low toxicity and eco-friendly properties (Hawadak et al., 2022). These characteristics make them a promising avenue in the development of new and more effective malaria treatments. The following paragraphs will detail various studies that showcase the potential of plant-based nanoparticles in versatile malaria management, highlighting their efficacy and multifaceted applications in both prevention and treatment.

### 3.1 Plant-based nanoparticle strategies to prevent malaria

Interrupting the mosquito life cycle is a critical strategy in managing mosquito-borne diseases. Various methods have been employed for vector control, including the use of synthetic insecticides, plant extracts, essential oils, nereistoxin from marine annelids, and synthesized NPs (Nalini et al., 2017; Vinoth et al., 2019). Protecting human hosts from mosquito-borne diseases can be achieved through two primary strategies: guarding against mosquito bites, and targeting mosquito larvae for control (Rajakumar and Abdul Rahuman, 2011; Karthigeyan and Premalatha, 2019; Esmaili et al., 2021). These dual approaches underscore the effectiveness of both personal protection measures and larval stage control in reducing the transmission of diseases by mosquitoes.

The development and implementation of vaccine platforms represent a promising and emerging pathway in the comprehensive management of malaria (Draper et al., 2018). We briefly discuss efforts in nanotechnology to improve three preventative measures (mosquito repelling, larval killing and vaccination) for the future eradication of malaria.

#### 3.1.1 Nanoformulations with repellent activity

The emergence of chloroquine-resistant strains of malaria has heightened interest in alternative strategies to combat the disease, such as the use of mosquito repellents. This shift in focus is underscored by the research of Khater et al. (2019), which highlights the growing importance of repellents in the fight against malaria, especially considering the reduced effectiveness of traditional antimalarial drugs like chloroquine. Mosquito repellents are substances designed to be applied to surfaces, altering the properties of the treated area and its surroundings to make them unattractive and inhospitable to mosquitoes (Khater et al., 2019).

Depending on the origin of raw materials, repellents can be divided into two distinct groups: synthetic and biological repellents. Synthetic repellents are derived from chemical compounds, while biological repellents are obtained from natural, biogenic sources (Karthigeyan and Premalatha, 2019).

Typically, insect repellents provide a vapour barrier that prevents mosquitoes from encountering human skin (Esmaili et al., 2021). Both chemical and natural repelling agents can be infused into textiles, leading to the development of mosquito-repellent textiles (Sheikh et al., 2021b). These textiles are increasingly recognized as an effective approach in the field, offering essential features for deterring mosquitoes. Such repellent methods are crucial for protecting humans from mosquito bites, which are responsible for the transmission of various mosquito-borne diseases including malaria, dengue, yellow fever and filariasis, as well as the discomfort of bite irritation (Rajakumar and Abdul Rahuman, 2011; Karthigeyan and Premalatha, 2019).

Common commercial mosquito repellents such as N, N-diethyl-meta-toluamide (DEET), dimethyl phthalate (DMP), and allethrin, although widely used, pose environmental and health risks at high exposure levels. These substances have been associated with various adverse effects including bioaccumulation, resistance to degradation, allergic reactions, asthmatic symptoms, skin irritations, cardiovascular and neurological disorders, dermatitis, and damage to synthetic fabrics and plastics. Given these potential hazards, there is a growing interest in the development and use of eco-friendly repellents as a safer alternative for protection against mosquito bites (Karthigeyan and Premalatha, 2019; Khater et al., 2019; Ahmad et al., 2023).

##### 3.1.1.1 Essential oils-based nanoformulations as repellents

The shift towards the development of green repellents has garnered increasing interest as a viable alternative to traditional synthetic repellents. Notably, essential oils extracted from various plants, such as *Ficus glomerata*, *Eucalyptus globulus*, and *Mentha piperita*, have shown promising repellent effects against *Anopheles stephensi* mosquitoes (Esmaili et al., 2021). This trend towards natural, plant-based repellents reflects a growing emphasis on eco-friendly and health-conscious solutions in mosquito-borne disease prevention. Unfortunately, the practical application of essential oils as repellents is hampered by their instability, susceptibility to degradation in the presence of light, oxygen and temperature, and their generally lower efficacy compared to synthetic insecticides. However, phyto-insect repellents, a nanotechnology-based incorporation of these essential oils into nanoformulations, such as nanoemulsions, solid lipid nanoparticles, polymeric nanoparticles and nanogels, presents a promising solution. These nanoformulations can stabilize the essential oils, enhance their dispersion, and potentially increase their efficacy as repellents (Barradas and de Holanda e Silva, 2021; Kelidari et al., 2021; Osanloo et al., 2022a; Osanloo et al., 2022b).

Moemenbellah-Fard et al. (2022) developed nanogels using essential oils from *Elettaria cardamomum* Malton and *Zataria multiflora* Boiss. The authors evaluated these nanogels for their complete protection times (CPT) against mosquitoes and compared the results with DEET, a standard repellent; the nanogels containing 2.5% *Z. multiflora* Boiss essential oil demonstrated superior effectiveness, with a CPT of 600 min, compared to DEET’s CPT of 242 min. In contrast, *E. cardamomum* Malton essential oil exhibited a lower efficacy, with a CPT of 63 min. The high efficiency of *Z. multiflora* Boiss essential oil is attributed to its major constituents, carvacrol and thymol, which have been previously reported to be effective against certain mosquito species. In another study, Sheikh et al. (2021b) developed a nanoemulsion using essential oils from *E. globulus* and *Syzygium aromaticum*. This nanoemulsion was then applied to cotton-polyester (30–70) fabrics using a spray drying procedure. The repellence efficiency of the formulated nanoemulsion was assessed through a mosquito repellence cage test. Remarkably, the nanoemulsion-treated textiles exhibited significantly enhanced protection, with a CPT of 285 min, while bulk essential oils offered protection times of less than 5 min. This substantial increase in efficacy demonstrates the potential of nanoemulsion-based repellents in textile applications.

The repellent effects and formulation description of various essential oil-based nanoformulations are summarized in Table 1.

**TABLE 1:** Description of essential oil loaded in nanoformulation-repellent properties.

Source of essential oils	Nanomaterial type	Nanomaterial composition	Droplet size (nm)	Target larvae species	Standard CPT	EO nanoformulation CPT	References
*Zataria multiflora Boiss*	Nanogel	EO/Tween^®^ 20: 7.5/2.5% w/v and Carboxymethyl cellulose (3.5%)	8	An. *Stephensi*	DEET 2.5% (242 min)	600 min (2.5% E.O.)	Moemenbellah-Fard et al. (2022)
*Elettaria cardamomum (L.) Malton*	Nanogel	EO/Tween^®^ 20: 7.5/2.5% w/v and Carboxymethyl cellulose (3.5%)	86			63 min (2.5% E.O.)	
*Syzygium aromaticum and Eucalyptus Globulus*	Nanoemulsion	E. globulus EO/*S. aromaticum* EO/Tween^®^ 80/Tween^®^ 20/propylene glycol/DW: 3%/2%/7%/7%/16%/65%	11.2–23.1	An. *stephensi*	NA	285 min (2.5% E.O.)	Sheikh et al. (2021a)
*Zataria multiflora*	Solid lipid nanoparticles	EO/Stearic acid/Span^®^ 60/Tween^®^ 80: 1%/4%/2%/4%	134	An. *stephensi*	NA	93 min (1% E.O.)	Kelidari et al. (2021)
*Elettaria cardamomum*	Polymeric nanoparticles	EO/Chitosan/Tween^®^20: 0.5%/0.25%/0.5%	121	An. *stephensi*	DEET 0.5% (62 min)	3–34 min (2.5% E.O.)	Sanei-Dehkordi et al. (2021)

Note: CPT, complete protection time. NA: not available; EO: essential oil; DEET: N, N-diethyl-meta-toluamide; DW: distilled water.

#### 3.1.2 Nanoformulations with larvicidal activity

Synthetic insecticides such as permethrin and dieldrin have been widely used, primarily due to their rapid action in controlling pests (Gandhi et al., 2018). However, their use raises significant concerns. On one hand, these chemicals pose threats to human health, non-targeted organisms, and the overall ecological balance. On the other hand, the extensive use of chemical insecticides has led to the development of resistant species, further complicating pest control efforts (Santhoshkumar et al., 2011; Lallawmawma et al., 2015; Santhosh et al., 2015; Deepak et al., 2018). These dual challenges underscore the need for more sustainable and ecologically sound pest management strategies.

**FIGURE 3:**
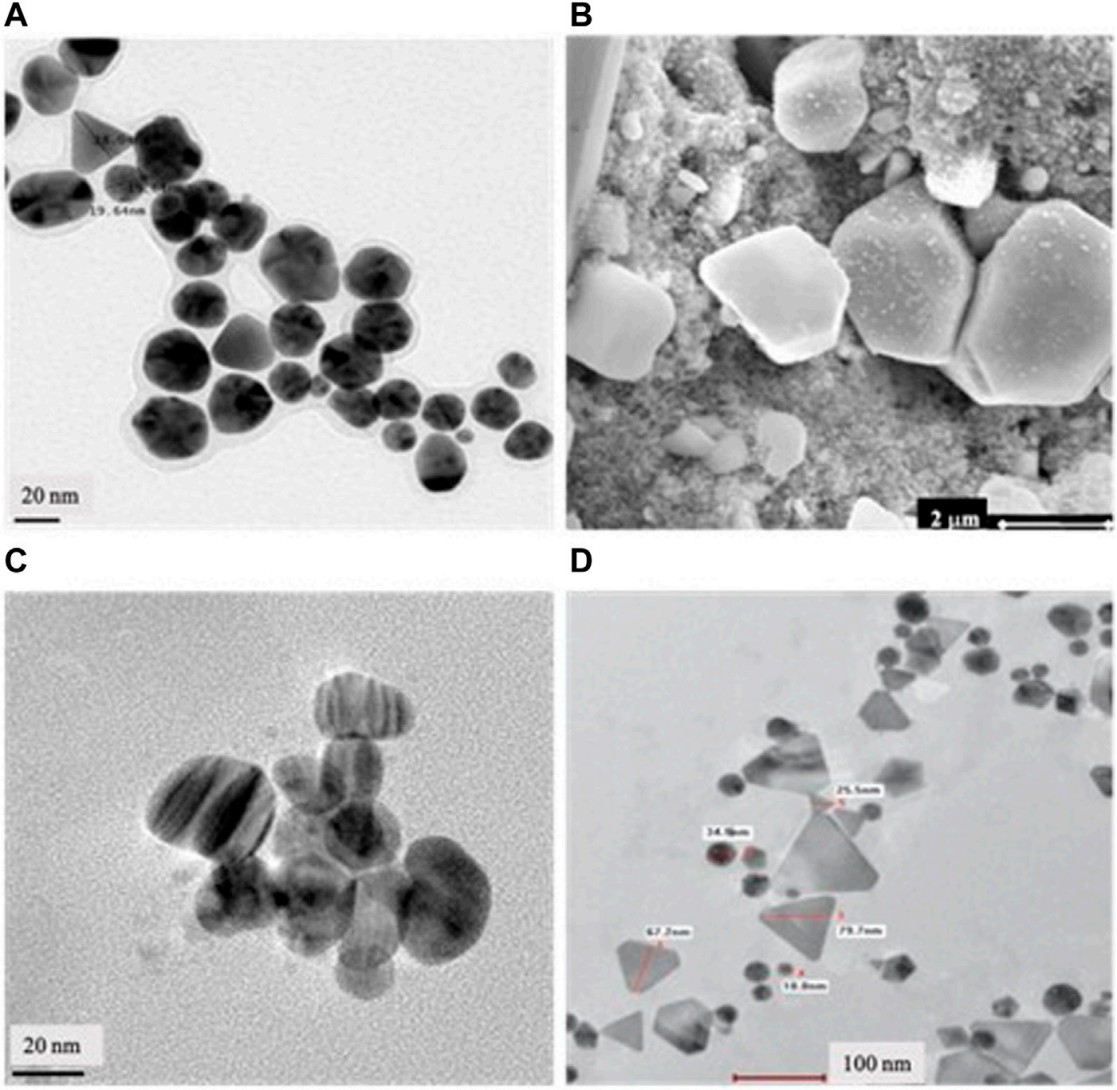
Illustrative microscopic appearance of biogenic nanoparticles with larvicidal properties. **(A)** Transmission Electron Micrograph (TEM) of biogenic gold NPs (AuNPs) from *Cymbopogon citratus*. **(B)** Scanning electron micrograph of biogenic silver NPs (AgNPs) from *Eclipta prostate*. **(C)** TEM of biogenic AgNPs from *Solanum nigrum*. **(D)** TEM of biogenic AuNPs from *Artemisinin vulgaris*. Reprinted from Murugan et al. (2015) (license number 5764290436824); Rajakumar and Abdul Rahuman (2011) (license number 5764290404752); Rawani et al. (2013) (license number 5764290360876) and Sundararajan and Ranjitha Kumari (2017) (license number 5764290220810) with the permission from Elsevier.

Plants are recognized as valuable sources of bioactive compounds with various properties, including mosquitocidal effects (Santhosh et al., 2015). The use of plant-based insecticides has become increasingly popular due to their cost-effectiveness and environmentally friendly nature. Consequently, plant-based nanoparticles is emerging as a promising alternative to synthetic insecticides, offering a more sustainable approach to pest control (Lallawmawma et al., 2015; Deepak et al., 2018; Vinoth et al., 2019). In the realm of nanotechnology, which occupies a forefront position in modern material science, the unique characteristics of nanoparticles (Figure 3), such as their nanometric sizes and high surface-to-volume ratios, present them as a viable alternative to conventional materials, especially where there are deficiencies in effectiveness or safety (Sowndarya et al., 2017; Hawadak et al., 2022).

Various types of plant-based nanoparticles have yielded effective mosquitocidal properties, either through their intrinsic larvicidal properties, as seen in metallic green nanoparticles, or by encapsulating larvicidal compounds, such as lipid-based nanoparticles infused with essential oils. The following paragraphs discuss these innovative approaches that showcase the versatility and potential of plant-based nanoparticles in mosquito control, particularly in targeting larval stages.

##### 3.1.2.1 Plant extract metallic NPs (MeNPs)

Plant-based MeNPs have shown a broad spectrum of applications in insect management. Their synthesis process is characterized by environmentally friendly features, including the use of biodegradable materials, nontoxic solvents and low-cost raw materials. These nanoparticles can be easily formulated on a large scale, as detailed in Table 2 (Sowndarya et al., 2017; Hawadak et al., 2022). Furthermore, plant-derived compounds can be utilized in the synthesis of effective mosquitocidal nanoparticles at low doses, offering an efficient and sustainable approach to mosquito control (Murugan et al., 2015).

**TABLE 2:** Larvicidal activity of plant extract-based MeNPs.

Plants	Plant part extracted	Type of MeNPs	Size (nm)	Shape	Mosquito name	MeNPs LC_50_	Crude aqueous extract LC_50_	References
*Clausena dentata*	Leaf	SeNPs	46.32–78.88	Spherical	*An. stephensi*	235.18–241.61 ppm 235.18–241.61 ppm	309.28–316.82 ppm 309.28–316.82 ppm	Sowndarya et al. (2017)
*Artemisia nilagirica*	Leaf	AgNPs	45	Spherical	*An. stephensi* and dengue vector	1.41–0.343 × 10^3^ ppm	7.22–2.24 × 10^3^ ppm	Nalini et al. (2017)
*Eclipta prostrata*	Leaf	AgNPs	35–60	Triangles, pentagons, and hexagons structures	*An. subpictus*	5.14 ppm	27.85 ppm	Rajakumar and Abdul Rahuman (2011)
*Annona muricata*	Leaf	AgNPs	20–53	Spherical	*An. stephensi*	13.58–16.97 ppm	34.33–85.83 ppm	Santhosh et al. (2015)
*Couroupita guinensis*	Flower	AuNPs	29.2–43.8	Spherical, Oval, triangular	*An. stephensi*	17.36–28.78 ppm	199.20–365.25 ppm	Subramaniam et al. (2020)
*Nelumbo nucifera*	Leaf	AgNPs	25–80	Circular	*An. subpictus*	0.69 ppm	11.82 ppm	Santhoshkumar et al. (2011)
*Momordica charantia*	Leaf	TiO_2_NPs	34.6–70.4	Irregular	*An. stephensi*	2.50–5.04 ppm	53.29 ppm	Gandhi et al. (2018)
*Solanum nigrum L*	Leaf	AgNPs	50–100	Spherical	*An. stephensi*	1.33–2.12 ppm	NA	Rawani et al. (2013)
*Solanum nigrum L*	Berries	AgNPs	50–100	Spherical	*An. stephensi*	1.54–2.44 ppm	NA	
*Citrus limon*	Leaf	PdNPs	1.9nm–4.8	Spherical	*An. stephensi*	7.21 × 104 (Minal and Prakash, 2018)72H	NA	Minal and Prakash (2018)

Note: LC_50_, Lethal concentration 50% mortality. NA: not available.

MeNPs synthesized using plant extracts may present a promising approach in mosquito vector management, potentially impacting malaria incidence significantly (Sowndarya et al., 2017; Hawadak et al., 2022; Nadeem et al., 2022). The insecticidal activity of secondary metabolites naturally synthesized by plants, such as alkaloids, essential oils, phenolics, steroids and terpenoids, has been well documented (Rajakumar and Abdul Rahuman, 2011; Hawadak et al., 2022). These compounds, most particularly phenolics known for their potent antioxidant properties, play a crucial role in reducing metallic ions to form and coat MeNPs, which leads to biogenic nanomaterials with enhanced, synergistic insecticidal activities (Santhosh et al., 2015). MeNPs from various plants, including *Clausena dentata*, *Artemisia nilagirica*, *Eclipta prostrata*, *Annona muricate*, *A. muricate*, and *Annona squamos*, as detailed in Table 2, have been found to be effective as larvicidal formulations against Anopheles species.

For example, Sowndarya et al. (2017) conducted a green synthesis of Selenium nanoparticles (SeNPs) using *C. dentata* leaf extract and evaluated their larvicidal activity against *An. stephensi*, *Culex quinquefasciatus* and *Aedes aegypti* larvae. The results indicated that the synthesized SeNPs exhibited significant larvicidal activity against *An. stephensi* at a low concentration, with an LC_50_ of 240.714 mg/L. The toxicity of these plant-based SeNPs on larvae and pupae was attributed to oxidative stress causing cell death by toxic reactive oxygen species (Nassar et al., 2023).

Another hypothesized mechanism of the larvicidal activity of SeNPs involves the denaturation of sulfur or phosphorus-containing compounds such as DNA. This denaturation could lead to reduced cellular membrane permeability, reduced ATP synthesis, and the denaturation of vital enzymes and organelles, ultimately causing loss of cellular function and cell death. Despite the promising potential of plant-based selenium nanoparticles, their path to commercialization remains largely unexplored. Before these plant-based SeNPs formulations can be commercialized, further *in vivo* studies are required to fully understand their safety and efficacy (Sowndarya et al., 2017). In the realm of biogenic insecticides, commercially available MeNPs, particularly silver nanoparticles (AgNPs) and gold nanoparticles (AuNPs), have garnered significant attention. Their unique properties and efficacy in pest control applications position them as prominent figures in the field of biogenic insecticide research and development. AgNPs are believed to possess the potential to penetrate larval membranes, potentially leading to significant biological effects. This penetration can result in the inactivation of enzymes and the generation of oxidative species within the midgut epithelial membrane of larvae, ultimately causing their death (Nalini et al., 2017). The potential of AgNPs as an effective tool in targeting and controlling mosquito larvae has been proven.

Santhoshkumar et al. (2011) evaluated the larvicidal activity of various extracts from *Nelumbo nucifera* Gaertn, including crude hexane, chloroformic, ethyl acetate, acetonic, methanolic and aqueous extracts, as well as synthesized AgNPs using aqueous leaf extract, against the fourth instar larvae of *Anopheles subpictus* Grassi and *C. quinquefasciatus* Say (Diptera: Culicidae).

AgNPs were formulated by reducing aqueous silver ions using the leaf extract of *N. nucifera*. AgNPs showed the highest larvicidal activity against *An. subpictus* larvae, followed by crude methanolic and aqueous extracts, with LC_50_ values of 0.69 ppm, 8.89 ppm, and 11.82 ppm, respectively. This indicates the superiority of AgNPs over regular crude plant extracts.

In another study, Nalini et al. (2017) synthesized AgNPs using the aqueous extract of *A. nilagirica* leaves. The larvicidal activity of these synthetized NPs larvicidal activity was evaluated against various developmental stages (I–IV instars and pupae) of *An. stephensi*. The LC_50_ values, calculated from the average mortality data of larvae, revealed that the synthesized AgNPs were more effective against developmental stages (I–IV instars) of *An. stephensi*, with LC_50_ values of 0.343%, 0.169%, 0.198%, and 0.141%, respectively, in contrast to 0.722%, 0.599%, 0.381%, and 0.224% for the aqueous leaf extract. This indicates that bioderived AgNPs using aqueous leaf extracts could be a viable, affordable, and eco-friendly approach for the development of biopesticides useful in malaria prevention.

In the same context, bioderived AuNPs from various plants have demonstrated significant insecticidal activity against mosquito larvae, even at very low dosages, making them promising tools for safer mosquito management (Sundararajan and Ranjitha Kumari, 2017). Murugan et al. (2015) synthesized AuNPs using the leaf aqueous extract of *Cymbopogon citratus*. The larvicidal activity of these *C. citratus* AuNPs against *An. stephensi* was found to be significantly higher than that of the crude extract. Specifically, the LC_50_ values for *C. citratus* AuNPs ranged from 18.80 to 41.52 ppm, compared to 219.32–471.36 ppm for the crude extract, indicating the enhanced efficacy of AuNPs in mosquito control. Overall, these findings underscore the enhanced efficacy of MeNPs in comparison to regular crude plant extracts. The superior larvicidal activity of MeNPs suggests their potential as a more effective alternative in mosquito control strategies.

##### 3.1.2.2 Essential oil-loaded nanoformulations with larvicidal properties

Beyond the inherently larvicidal MeNPs, a diverse range of nanoformulations has been developed for encapsulating and controlling the delivery of plant-derived larvicidal agents. These nanoformulations serve as carriers, enhancing the dispersion, bioavailability, efficacy and specificity of larvicidal compounds. For example, Sanei-Dehkordi et al. (2022) reported the larvicidal activities of liposomes made up of a mixture of egg yolk lecithin (2.5% w/v), Cholesterol (0.5% w/v), Tween 20 (1.0% w/v), and essential oils (2.0% w/v) from *Citrus aurantium*, *Citrus limon*, *Citrus sinensis* and crude limonene extracts. These liposomes, particularly those containing limonene and limonene-rich essential oils, demonstrated significantly higher toxicity (almost 10 times for *C. aurantium*) compared to free essential oils against *An. Stephensi*, which underscores the crucial role that liposomes play in enhancing the delivery of essential oils. The authors observed that *C. aurantium* liposomes exhibited the most potent larvicidal activity against *An. Stephensi*, with an LC_50_ value of 6.63 μg/mL, suggesting their potential as an alternative to synthetic insecticides. By encapsulating these oils, liposomes improve their stability, efficacy and potential for targeted application in larvicidal treatments. In addition to liposomes, other types of nanoformulations such as nanoemulsions and nanogels have also shown significant potential in enhancing the larvicidal activities of essential oils (Sanei-Dehkordi et al., 2022). Additional examples of nanoformulations containing essential oils from various plants, known for their larvicidal effects, are detailed in Table 3. These nanoformulations showcase the diversity and efficacy of plant-derived essential oils in mosquito control, when enhanced through nanotechnology.

**TABLE 3:** Essential oils-containing nanoformulations with larvicidal effect against malaria vector larvae.

Plant name	Nanomaterial type	Formulation composition	Size	Target larvae species	LC_50_ of non-formulated E.O.	LC_50_ of E.O. loaded in NPs	References
*Artemisia annua*	Liposomes	Egg lecithin/Chol/Tween^®^ 20/E.O: 3.0/0.5/2.0/2.0%w/v	137 nm	*An. Stephensi*	NA	90 μg/mL	Sanei-Dehkordi et al. (2023)
*Artemisia dracunculus*	Liposomes	Egg lecithin/Chol/Tween^®^ 20/E.O: 3.0/0.5/2.0/2.0%w/v	151 nm	*An. Stephensi*	NA	23 μg/mL	
*Artemisia sieberi*	Liposomes	Egg lecithin/Chol/Tween^®^ 20/E.O: 3.0/0.5/2.0/2.0%w/v	92 nm	*An. Stephensi*	NA	140 μg/mL	
*Artemisia dracunculus*	Nanoemulsion	E.O/Tween^®^ 20/Water	152 nm	*An. Stephensi*	NA	13.5 (7–25) µg/mL	Osanloo et al. (2022b)
*Artemisia dracunculus*	Nanogel	E.O/Tween^®^ 20/Water/Carboxycellulose	NA	*An. Stephensi*	NA	6.6 (2–19) µg/mL	
*Citrus aurantium*	Liposomes	Egg lecithin/Chol/Tween^®^ 20/E.O: 2.5/0.5/1.0/2.0%w/v	52 nm	*An. Stephensi*	62.49 μg/mL	6.63 μg/mL	Sanei-Dehkordi et al. (2022)
*Citrus limon*	Liposomes	Lecithin/Chol/Tween^®^ 20/E.O: 2.5/0.5/1.0/2.0%w/v	67 nm	*An. Stephensi*	13.87 μg/mL	6.8 μg/mL	
*Citrus sinensis*	Liposomes	Lecithin/Chol/Tween^®^ 20/E.O: 2.5/0.5/1.0/2.0%w/v	53 nm	*An. stephensi*	12.41 μg/mL	9.6 μg/mL	

Note: LC_50_, Lethal concentration 50% mortality. NA: not available; EO: essential oil; Chol: Cholesterol.

#### 3.1.3 Vaccine candidates from plant virus-like nanoparticles

The emergence of chloroquine-resistant Plasmodium strains, the lack of alternatives to primaquine, and the global rise in temperatures have compounded the challenges in effective malaria management. This has led to numerous studies exploring plants used in traditional medicines for the development of new drugs and vaccine formulations (Clemente and Corigliano, 2012; Lebel et al., 2015; Kekani and Witika, 2023). The difficulty in effectively treating malaria has highlighted the limitations of traditional vaccines, emphasizing the need for novel vaccine platforms in the fight against this disease. Recently, there has been a growing interest in utilizing plant virus-like nanoparticles and recombinant plant viruses in the research and development of new, safe, and effective adjuvants and vaccines for infectious diseases, including malaria (Lebel et al., 2015).

Malaria transmission-blocking vaccines (TBVs) have emerged as a promising approach to reducing the transmission of the disease. These vaccines work by inducing the production of antibodies in the human host, when a vaccinated individual is bitten by a mosquito, these antibodies are ingested during the blood meal (Blagborough et al., 2016). They then act by preventing the development of the parasite within the mosquito’s midgut. This is achieved by binding to surface proteins of the sexual stages of the parasite, thus impeding further transmission. One of the primary targets of these TBVs is the P25 protein, which is expressed by the parasite’s gametes, zygote and ookinete stages. The targeting of P25 has been a focal point in the development of effective malaria TBVs (Carter et al., 2000; Saxena et al., 2007).

While the P25 protein is a key target for TBVs, the production of recombinant P25 protein that maintains the native conformation presents significant challenges. This complexity is due to its intricate structure, which includes epidermal growth factor (EGF)-like motifs, multiple cysteine residues, and a complex tertiary structure (Sala et al., 2018; McCaffery et al., 2019). Consequently, the use of plants in the formulation of vaccine platforms offers a cost-effective and scalable alternative. Plant-based systems have the potential to express the native conformation of P25 proteins of *Plasmodium* species, which has been a limitation in small-scale studies. This approach could revolutionize the production of effective malaria TBVs, making them more accessible and feasible for widespread use (Farrance et al., 2011).

In a notable study by Blagborough et al. (2016), a Tobacco Mosaic Virus (TMV)-based hybrid expression vector, pGRD4, was utilized to produce the recombinant *P. vivax* P25 protein in *Nicotiana benthamiana*, fused to a modified lichenase carrier protein. The vaccine candidate, named Pvs25-FhCMB, underwent efficiency evaluation through a head-to-head comparison. This involved immunizing mice with the recombinant form of Pvs25-FhCMB, in combination with two clinically relevant adjuvants: Alhydrogel (a common aluminium hydroxide wet gel suspension) and Abisco-100 (a non-toxic saponin-based adjuvant). The results were then compared with immunization using a leading adenoviral vaccine platform to assess transmission-blocking (TB) efficiency. The mice were then infected with *Plasmodium berghei* Pvs25DR3. Immunization with the adenoviral vaccine boosted with Pvs25-FhCMB/Abisco-100 showed the highest efficacy, both in terms of intensity and prevalence reduction, followed by Pvs25-FhCMB/Abisco-100 and Pvs25-FhCMB/Alhydrogel. The mean reduction in intensity/prevalence was 74.5%/68.3%, 65%/54%, and 56.6%/51.6%, respectively. This study not only demonstrated the potential for anti-malarial TBV production using this approach but also highlighted that the combination with an adenoviral vaccine platform resulted in maximal efficiency.

In a breakthrough study by Chichester et al. (2018), a novel malaria TBV was developed, targeting proteins specific to the sexual stages of *P. falciparum* present in the mosquito midgut. This vaccine candidate, named Pfs25 VLP-FhCMB, was engineered as a chimeric non-enveloped virus-like particle. It contained the recombinant Pfs25 antigen from *P. falciparum* fused to the Alfalfa Mosaic Virus coat protein. The Fraunhofer United States Center for Molecular Biotechnology (FhCMB) developed this vaccine using *N. benthamiana* as the expression system, with *Rhizobium radiobacter* (formerly *Agrobacterium tumefaciens*) for transfection and Alhydrogel as the adjuvant. Pfs25 VLP-FhCMB was demonstrated to be safe at all tested doses in healthy human adult volunteers, with no vaccine-related serious side effects or adverse events reported. However, while the Pfs25 VLP vaccine successfully induced an immune response in the participants, the transmission reduction activity of the generated antibodies did not reach significant levels. This outcome suggests the need for alternative vaccine adjuvant formulations or combinations to enhance Pfs25 VLP efficacy (Lebel et al., 2015).

### 3.2 Plant-based nanoparticles for malaria treatment

#### 3.2.1 Metallic nanoparticles from plant extracts

Green synthesized nanoparticles have garnered attention for their potential antiplasmodial activity. The notable properties of certain metal oxide and metallic nanoparticles, including silver, iron, nickel, gold, copper oxide and zinc oxide, such as magnetic characteristics, ease of separation, large surface area and minimal side effects have made them increasingly attractive in the development of a wide range of antimalarial formulation (Nadeem et al., 2022). As listed in Table 4, various plants have been employed in the reduction and stabilization (capping) of the metallic ions leading to the eco-friendly synthesis of anti-plasmodial metallic nanoparticles. For example, Hawadak et al. (2022) utilized the aqueous extracts of *Azadirachta indica*, a plant known for its antimalarial properties, for the synthesis of AgNPs from both leaves and bark. These AgNPs were evaluated for their anti-plasmodial activity against *P. falciparum* strains, both chloroquine-sensitive (CQs) 3D7 and chloroquine-resistant (CQr) RKL9, as well as for their hemocompatibility and stability. Notably, the AgNPs derived from leaves exhibited superior antimalarial activity, with an IC_50_ value of 7.87 μg/mL against the RKL9 strain and 8.10 μg/mL against the 3D7 strain. Both leaves and bark AgNPs demonstrated enhanced antiplasmodial activity compared to the crude aqueous extracts of *A. indica* (with IC_50_ = 33.97 μg/mL and 49.64 μg/mL against 3D7; 46.82 μg/mL and 54.10 μg/mL against RKL9, respectively). However, AgNPs synthesized from *A. indica* exhibited increased hemolytic activity on fresh human red blood cells (RBCs). This underscores the need for further investigation into the complex interactions between AgNPs and RBCs to enhance their hemocompatibility and reduce potential toxicity.

**TABLE 4:** Antiplasmodial activity of plant extract-based MeNPs.

Plants	Plant part extracted	Type of MeNPs	Size (nm)	Shape	Target Plasmodium strains	Antiplasmodial activity	References
*Andrographis paniculata* Nees	Leaf	AgNPs	35–55	Spherical	*P. falciparum* parasites present in patient blood	Parasitemia at 100 μg/mL = 83%	Panneerselvam et al. (2011)
*Rhazya stricta*	Leaf	ZnONPs	70–90	Spherical	Plasmodium parasites present in patient blood	IC_50_ = 3.41 μg/mL	Najoom et al. (2021)
*Leucaena leucocephala*	Leaf	CdONPs	36–57	Spherical	*P. falciparum*	IC_50_ = 0.95 μg/mL	Savale et al. (2017)
*Nephrolepis exaltata*	*NA*	FeONPs	16	Spherical	Plasmodium parasites present in patient blood	Parasite Inhibitory Concentration = 62.0%	Nadeem et al. (2022)
*Crataegus ambigua*	Leaf	AgNPs	32	Spherical	*P. falciparum asexual parasites*	Parasite Inhibitory Concentration at 10µ g/mL = 96.2%	Ojemaye et al. (2021)
	Fruit	AgNPs	32	Spherical	*P. falciparum* asexual parasites	Parasite Inhibitory Concentration at 10 μg/mL = 97.1%	
*Azadirachta indica*	Leaf	AgNPs	20	Spherical	*P. falciparum RKL9* strains	IC_50_ = 7.87 μg/mL	Hawadak et al. (2022)

Note: IC_50_: Half-maximal inhibitory concentration; NA: not available.

In another study, iron oxide nanoparticles (FeONPs) from *Nephrolepis exaltata* (at a concentration of 25 μg/mL) exhibited significantly higher antiplasmodial efficiency than both crude plant extract and FeCl_3_·6H_2_O used as a precursor, with mean parasite inhibitory concentration values of 62%, 35%, and 23%, respectively (Nadeem et al., 2022). The phytochemical analysis of *N. exaltata* revealed the presence of compounds such as saponins, steroids, tannins, alkaloids and phenols, which served as reducing and capping agents in the synthesis of FeONPs. Considering the ISO 10993–5: 2009 limit (greater than 70%), FeONPs at 25 μg/mL are deemed non-toxic, displaying a cell viability percentage of 78%. These findings suggest that FeONPs from *N. exaltata* hold great potential for the development of new biocompatible antimalarial drugs.

#### 3.2.2 Soy lecithin-based nanoformulations

The majority of antimalarial drugs under development are hydrophobic, characterized by extensive biodistribution volumes and poor plasma solubility, which results in low accumulation in RBCs. This limitation adversely affects the drug’s efficacy. As a solution, lipid nanoparticles have emerged as promising carrier systems.

They are recognized for their potential to improve the pharmacokinetics properties of antimalarial drugs, including enhancing solubility and ensuring more effective drug delivery to target sites (Memvanga and Nkanga, 2021).

Numerous natural lipids, including soybean lecithin, have been utilized in the creation of NPs which have demonstrated considerable success in significantly optimizing the therapeutic efficiency of antimalarial agents (Memvanga and Nkanga, 2021). Soybean lecithin has been used to make various lipid-based NPs (including solid lipid NPs, liposomes, and nanocapsules) that have proven the potential to enhance drug delivery, solubility and stability, thereby improving the overall effectiveness of antimalarial treatments. For instance, Ramteke et al. (2018) developed solid lipid nanoparticles as a carrier for artemether, intended for intranasal administration. The formulation utilized soy lecithin soya as the surfactant and poloxamer 407 as the co-surfactant, with the lipid phase comprising glyceryl monostearate and artemether. The efficacy of this intranasal delivery system was evaluated *in vivo* on Wistar rats. The optimal SLNs containing 0.077 mg of artemether resulted in a higher concentration of the drug in cerebrospinal fluid (CSF) compared to a free drug suspension containing a 0.154 mg dose given orally.

In a manner akin to SLNs, liposomes made from soybean lecithin have also demonstrated effectiveness in the delivery of antimalarial drugs. These soybean lecithin-based liposomes enhance the bioavailability and targeted delivery of the drugs, thereby potentially increasing their therapeutic efficacy. As an example, Rajendran et al. (2016) developed liposomes using soya phosphatidylcholine (SPC) and cholesterol (Chol), incorporating either stearylamine (SA) or phosphatidic acid (PA), and loaded them with monensin for their *in vitro* antimalarial activity evaluation against *P. falciparum* 3D7.

The study found that SA liposome exhibited the most potent antimalarial activity against *P. falciparum* 3D7, followed by SPC: Chol liposome, PA liposome and free monensin, with IC_50_ values of 0.74, 1.11, 2.98, and 3.17 nM, respectively. The superiority of SA liposomes was attributed to their enhanced adsorption or binding to infected erythrocytes, which are characterized by a high level of phosphatidylserine (PS) on their outer membrane; the affinity was hypothesized to be due to the electrostatic interaction between the positively charged SA and the negatively charged surface of the infected RBCs. This interaction could potentially disrupt the membrane and lead to cell death (Rajendran et al., 2016; Vassoudevane et al., 2023).

By enhancing the delivery of antimalarial drugs, soybean lecithin NPs have also shown great potential in improving the safety profiles of various drug molecules, including Halofantrine.

Halofantrine hydrochloride, known for its efficacy against *P. falciparum*, particularly with the emergence of chloroquine-resistant strains, has been a focus of research. However, Halofantrine is associated with serious cardiotoxic effects, including the prolongation of the QT interval, a concern also associated with the cardiac side effects of quinine and mefloquine. By allowing for controlled release and targeted delivery and reducing the systemic exposure of the heart to the drug, soybean lecithin NPs have proven great potential in mitigating the cardiotoxic effects of halofantrine (Mosqueira et al., 2004). In the same study, authors developed nanocapsules using soy lecithin (0.75% wt/vol) as the hydrophobic surfactant and poloxamer 188 as the hydrophilic surfactant. The oil phase consisted of miglyol 810 (2.5% wt/vol), and poly (D, L-lactide) (PLA) homopolymer was used as the oily core of nanocapsules. The halofantrine nanocapsule formulation showed no toxic effects after intravenous administration for doses up to 100 mg/kg. Moreover, the nanocapsules formed improved parasite management 48 h post-treatment. The study suggested that nanocapsule formulations could offer a more favourable halofantrine plasma profile, potentially allowing for a reduction in the required intravenous dose.

#### 3.2.3 Plant-based oil nanoformulations

Plant-derived oils, as a superior alternative to synthetic materials, have been increasingly utilized in the formulation of nanocapsules for the encapsulation of antimalarial drugs. This approach has proven to significantly enhance the pharmacological profiles of these drugs, offering improved efficacy, stability and bioavailability. In their research, Gomes et al. (2018) utilized *Curcuma aromatica* oil (CO) as the oil-based core for developing Eudragit RS100 nanocapsules, coated with polysorbate for the encapsulation of quinine. It was found that nanoencapsulation of quinine in the Eudragit RS100 and CO formulation improved the drug’s photostability and efficiency for the nano-encapsulated quinine. Compared to 28.8% for free quinine, the photodegradation percentage was significantly reduced to 5.1%. Additionally, the suppression percentage and mean parasitaemia percentage were 8.49% and 61% for the nano-encapsulated formulation, in contrast to 4.88% and 41% for free quinine, indicating enhanced therapeutic effectiveness. The impact of cationic nanocapsules, specifically those made with Eudragit RS100, has also been explored. The positive charge of Eudragit RS100 nanocapsules was attributed to the quaternary ammonium group present in the polymer. This characteristic positively influences the zeta potential of the nanocapsules, thereby enhancing electrostatic interactions between the drug formulation and cell membranes. Such interactions are key to improving the biological perf (Yurtdaş-Kırımlıoğlu and Görgülü, 2021). Studies have proved the stability and viability of vegetable oil core nanocapsules made of Eudragit RS100. Brazil nut, sunflower seed, olive, rose-hip, grape seed and carrot oils presented a higher reversible creaming of the nanoparticles due to their lower density compared to capric/caprylic triglycerides; and could be alternatives to standard triglycerides for composing the oily core of polymeric nanocapsules (Contri et al., 2013; Santos et al., 2021).

Recent advancements in antimalarial drug formulation have also been focused on improving the solubility and bioavailability of poorly water-soluble plant-derived antimalarial drugs. This is particularly applicable to curcumin (diferuloylmethane) from *Curcuma longa* and artemisinin from *A. annua*. Curcumin and artemisinin are both known for their antimalarial activity and poor water solubility, which underlines their low oral bioavailability and rapid elimination from the body (Malik and Mukherjee, 2014; Gao et al., 2023). To address these issues, the self-micro emulsifying drug delivery system (SMEDDS) has been introduced as a promising solution. SMEDDS, composed of a mixture of oil, surfactant (potentially with a co-surfactant), and the drug, can form a microemulsion in the gastrointestinal fluid, enhancing gastrointestinal motility after oral administration. SMEDDS has been recognized as one of the most effective approaches to improving the solubility and oral absorption of poorly water-soluble drugs. In the quest to improve the solubility of curcumin and artemisinin, studies by Shah et al. (2017) revealed that these compounds exhibited better solubility in corn oil, followed by olive oil. However, challenges were encountered with both oils. Corn oil led to the formation of a turbid emulsion upon dilution, resulting in drug precipitation, while olive oil tended to form a separate phase. These limitations necessitated the exploration of an alternative oil, leading to the selection of oleic acid, which demonstrated improved solubility properties (4.4 mg/3 mL for curcumin and 73.4 mg/3 mL for artemisinin). In terms of surfactants and co-surfactants, Tween 80 and PEG-400 were identified as the most effective in solubilizing these compounds. This combination resulted in the development of a SMEDDS for curcumin and artemisinin with attractive characteristics: an average droplet size of 150.7 nm, with the potential of loading up to 63.81% of curcumin and 54.91% of artemisinin which could be dissolved in pH 1.2 media within 1.5 h. This indicates a significant improvement in the solubility and potential bioavailability of these antimalarial drugs in the gastrointestinal environment, however, data demonstrating the actual impact of such SMEDDS on this antimalarial drug bioavailability is pending.

#### 3.2.4 Protein-based nanoparticles for malaria

The utilization of albumin for nanoformulation of plant-derived antimalarial drugs, such as artemisinin and its derivatives, has been explored. Although artemisinin-based combination therapies have become cornerstone treatments for malaria, there are still several challenges such as poor water solubility, low bioavailability and a short half-life, which may preclude their maximum potential from unfolding fully (Yaméogo et al., 2020). Human serum albumin (HSA) has been proposed as a viable antimalarial drug carrier due to its targeting potential towards malaria-infected erythrocytes, as well as excellent availability, biodegradability, non-toxicity and non-immunogenicity, which make it an excellent option for antimalarial drug delivery (Boateng-Marfo et al., 2018; Bhide et al., 2023).

Ibrahim et al. (2014) developed an HSA-bound, water-soluble nanoparticulate formulation of artemisinin (ART) for injection, addressing the drug’s physicochemical and biopharmaceutical challenges. This formulation demonstrated high entrapment efficiency (97.5%) and exhibited superior *in vitro* antimalarial activity against the chloroquine-resistant *P. falciparum* strain FcB1 compared to free artemisinin and chloroquine, with IC_50_ values of <3.5 nM, 11.4 nM, and 123.5 nM, respectively. Importantly, the use of albumin-bound nanoparticles enabled the intravenous administration of ART without the need for organic solvents or co-solvents, achieving 100% bioavailability. HSA’s strong binding to artemisinin and its derivatives through thiol and amino groups further enhances the drug’s antimalarial activity (Sidhaye et al., 2016). The development of HSA-based nanoparticles represents a significant advancement in the selective targeting of antimalarial drugs, offering a promising and more effective approach to treating malaria.

#### 3.2.5 Nanogels for antimalarial drug delivery

Hydrogel nanoparticles (*a.k.a.* nanogels) have emerged as a viable solution to overcome challenges associated with plant-derived antimalarial agents, including curcumin. Curcumin exhibits poor water solubility, which poses significant challenges to its absorption in the gastrointestinal tract, and consequently, its oral bioavailability (Lv et al., 2013; Santos et al., 2021; Onyenji, 2022). By offering hydrophilicity, high water absorptivity, versatility, flexibility and biocompatibility, nanogels have proven good potential to facilitate prolonged circulation time and enable both active and passive targeting of poorly water-soluble drugs (Amin et al., 2023). Dandekar et al. (2010) developed nanogels using a blend of hydroxypropyl methylcellulose and polyvinylpyrrolidone, aiming to enhance the absorption and extend the clearance of curcumin by potentially evading the reticuloendothelial system. The optimization process varied the hydroxypropyl methylcellulose to curcumin ratio from 0.5:1 to 3:1 and incorporated various surfactants, including TPGS (D-α-Tocopheryl polyethylene glycol 1,000 succinate), Tween-80, Tween-20, Cremophor^®^ RH 40, Pluronic^®^ F68, and Pluronic^®^ F127, with the curcumin ratio ranging from 0.5:1 to 3:1. The optimal nanoformulation, made up of a hydroxypropyl methylcellulose to curcumin ratio of 2:1 and a Pluronic^®^ F68 to curcumin ratio of 2:1, was characterized by particle sizes of 98 nm, a polydispersity index of 0.31, and an encapsulation efficiency of 72% for curcumin.

In the same study, researchers also investigated *in vivo* antimalarial efficacy (using male Swiss mice) and acute toxicity (using Organization of Economic Cooperation and Development guidelines 425 and 407) of the formulated NPs. A significant (*p* < 0.05) improvement in the parasitaemia compared to the curcumin control was observed for nanoparticles at a dose equivalent to 25 mg/kg body weight. The acute toxicity test demonstrated the safety of the nanoparticles at a dose equivalent to 2000 mg/kg body weight. This optimized formulation showed promise as an adjuvant system for antimalarial therapy, potentially aiding in recrudescence prevention and/or reducing the dosage of standard antimalarial drugs (Dandekar et al., 2010; Amin et al., 2023).

## 4 Surface-decorated plant-based nanoparticles for malaria treatment

**FIGURE 4:**
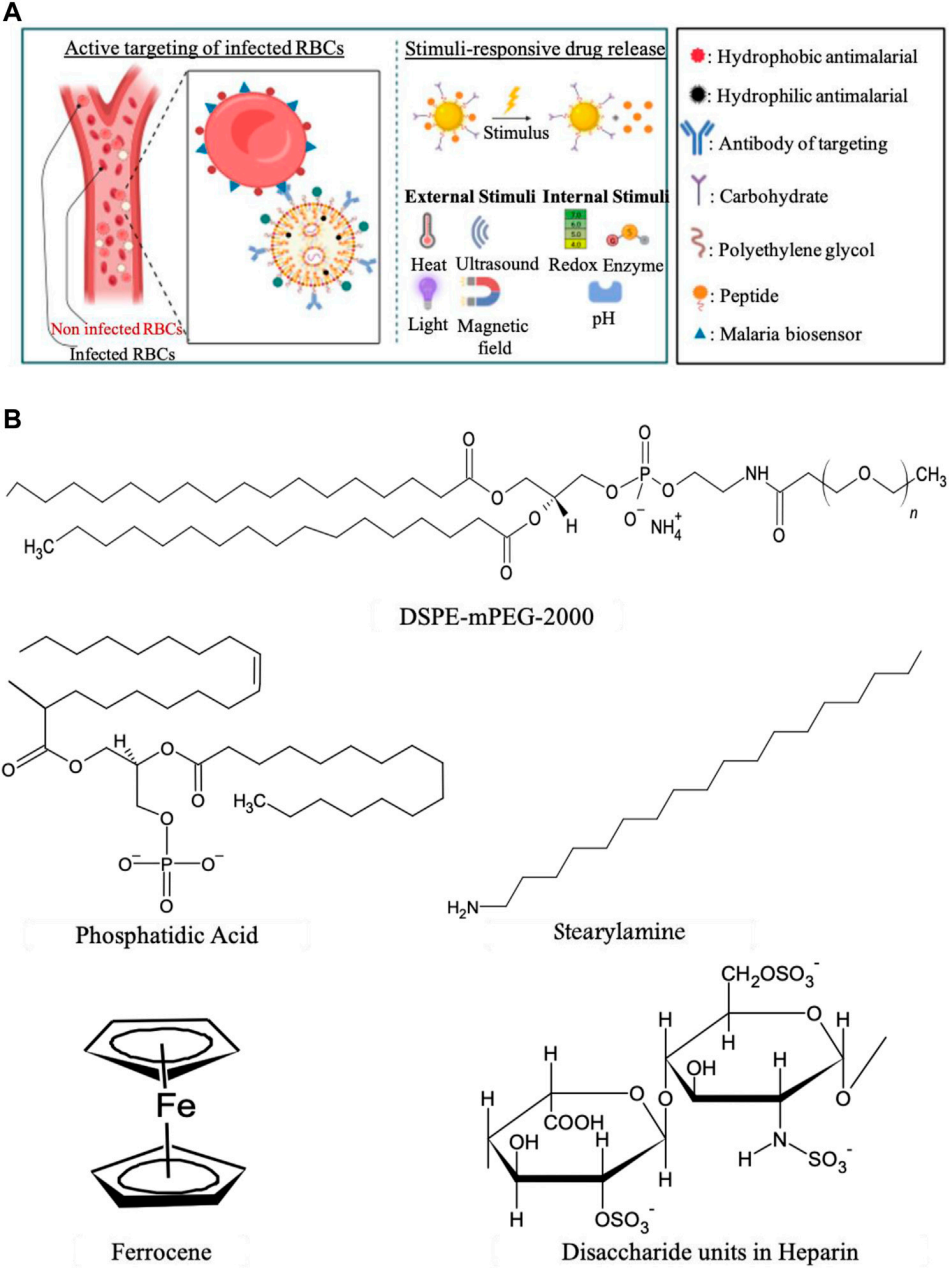
Illustration of nanoparticles targeting infected red blood cells and different features that can be used to decorate surface NPs to target passively or actively malaria biomarkers **(A)** and structures of chemical entities used as ligands targeting infected red blood cells **(B)**. BioRender (online) and ChemSketch 2021.2.1 version C35E41 were used to create the structures in the two panels, respectively.

Nanocarriers, used to enhance the pharmacokinetics of drugs, can be surface decorated with various molecules to achieve improved circulation in the bloodstream. These include polyethylene glycol for extended circulation, binding agents, or biomarker recognition agents for specific targets, as depicted in Figure 4 (Nisini et al., 2018). Once the active ingredient is encapsulated or attached to the nanocarrier, it is crucial to ensure cargo release to complete targeted delivery. For this, nanoparticles can be functionalized with stimuli-responsive agents that respond to specific triggers such as heat, ultrasound, pH, redox, light, magnetic fields or to enzyme activity, leading to controlled drug release (Tawfik et al., 2021), as illustrated in Figure 4A. In the case of malaria, maintaining the presence of the drug in the bloodstream is essential for its effective interaction with infected red blood cells and plasmodium membrane. Passive and active targeting techniques are utilized to direct the drug to infected red blood cells or hepatocytes, which are the sites of the disease (Mosqueira et al., 2004).

Passive targeting is achieved using conventional nanocarriers or surface-modified long-circulating nanocarriers, such as PEGylated nanocarriers (Mosqueira et al., 2004; Memvanga and Nkanga, 2021).

In contrast, active targeting involves the surface modification of nanocarriers with specific ligands, such as glycolipids, carbohydrates, peptides, proteins, or antibodies (Salahpour Anarjan, 2019; Memvanga and Nkanga, 2021) as shown in Figure 4B. We delve into the realm of surface-decorated nanocarriers specifically designed for targeted antimalarial drug delivery. These nanocarriers are engineered with surface modifications to enhance the delivery of antimalarial drugs directly to the infected cells (erythrocytes or hepatocytes), thereby improving the efficacy and reducing potential side effects of the treatment.

### 4.1 Ligand-decorated lipid nanoparticles

#### 4.1.1 Surface-modified liposomes

When introduced into the body, conventional liposomes are often rapidly detected as foreign entities by the immune system, leading to their swift degradation. This shortens their circulation time in the blood and results in the accumulation of degraded liposome in the liver and spleen (Nsairat et al., 2022). One strategy to manage this issue is to reduce the size of the liposomes. Liposomes with a diameter of less than 20–30 nm have been shown to accumulate less in vital organs. Their smaller size enables them to pass more easily through the capillary walls and re-enter the bloodstream via diffusion, whereas larger liposomes face challenges due to their size-limited permeability (Le et al., 2019).

To address these limitations, surface-modified liposomes have been developed. These include PEGylated liposomes, which have extended circulation times, ligand-targeted liposomes, which can actively target specific cells or tissues, and multifunctional liposomes, which combine various features for optimized delivery. Such modifications offer promising solutions to overcome the challenges associated with conventional liposomes, making them more suitable for targeted drug delivery (Le et al., 2019).

##### 4.1.1.1 Surface-decorated liposomes for passive targeting

Liposomes used for malaria treatment primarily act within the bloodstream, necessitating prolonged circulation time for maximum efficacy. One effective strategy for achieving this is surface stabilization using hydrophilic and biocompatible polymers, such as polyethylene glycol (PEG). Surface modification involves covalently conjugating or coating the surface of liposomes with biopolymers. This modification not only prevents the absorption of serum proteins by the liposome but also extends their circulation time in the blood. A key advantage of PEGylated liposomes is their reduced recognition by macrophages and the reticuloendothelial system (RES), leading to a decrease in phagocytic clearance (Isacchi et al., 2011; Kirtane et al., 2021; Memvanga and Nkanga, 2021).

For example, amphiphilic γ-cyclodextrin nanoformulations encapsulating artemisinin have shown poor blood residency due to severe accumulation in hepatosplenic regions. The formulation of these amphiphilic γ-cyclodextrins with PEGylated phospholipids (such as PEG1500-stearate, DMPE-PEG2000, polysorbate 80 or PEG4000-stearate) at a ratio of γ-CD-C10/PEG of 3:1 and bio-esterified γ-cyclodextrins fatty esters (γ-CD-C10), have resulted in liposomal systems which exhibited extended circulation time in the bloodstream (Yaméogo et al., 2014; Yaméogo et al., 2020). To assess the plant-derived antimalarial drug delivery potential, Yaméogo et al. (2020) tested artemisinin-loaded nanoparticles intravenously in healthy rats, using doses of 1.5 mg/kg or 2 mg/kg. Compared to a reference solution of artemisinin in 10% ethanol, the nanosystems maintained artemisinin plasma concentrations above therapeutically significant levels for extended periods of 8 h for DMPE-mPEG2000/γ-CD-C10, *versus* only 2 h for the ethanol solution. This highlights the potential of PEGylated liposomal systems to be a promising approach to improving the bioavailability and therapeutic efficacy of plant-derived antimalarial agents. Similarly, PEGylation has been used to improve the pharmacological performance of liposomes made of plant-derived lipids, such as crude soybean lecithin. Soy lecithin liposomes have been utilized as carrier systems for delivering monensin, an anticoccidial drug that exhibits antimalarial activity against *P. falciparum in vitro* and *Plasmodium vinckei* Petteriand, *Plasmodium chabaudi in vivo*, as well as in combination with artemisinin (Rajendran et al., 2016).

This research focused on assessing Monensin’s antimalarial activity in lipid formulations comprising soya phosphatidylcholine (SPC) and cholesterol (Chol), with either stearylamine (SA) or phosphatidic acid (PA), and varying concentrations of distearoyl phosphatidylethanolamine methoxy-polyethylene glycol 2000 (DSPE-mPEG-2000). In the same study it was found that incorporating different densities of DSPE-mPEG 2000 on the liposomes’ surfaces enhanced the antimalarial activity of monensin in SPC:Chol and PA liposomes, with the most notable improvement in SPC: Chol liposomes modified with 5 mol% PEG 2000 (IC_50_ = 0.39 nM), followed by PA liposomes (IC_50_ = 0.81 nM for 5 mol% PEG 2000). Surface modification of SA liposomes with DSPE-mPEG 2000 resulted in prolonged circulation in the blood but did not affect the parasite’s growth, which is probably due to excessive PEGylation decreasing cellular uptake and therefore reducing the formulation efficiency.

Beyond soybean lecithin, other plant-derived excipients such as Nutriose^®^ FM06, a water-soluble, fibre-rich branched dextrin with prebiotic activity, are being explored for vesicular nanocarriers in antimalarial drug delivery. When used in soy phosphatidylcholine vesicles, Nutriose FM06^®^ enriches the formulation, creating a vesicular system (namely nutriosomes) encapsulating various plant-derived drugs such as artemisinin, curcumin and quercetin, with entrapment efficiencies as high as 19%, 65% and 49%, respectively (Manconi et al., 2019; Fulgheri et al., 2023).

The antiplasmodial efficiency of artemisinin-loaded nutriosomes in testing was lower than that of artemisinin solution, with IC_50_ values of 0.070 μg/mL and 0.006 μg/mL, respectively. However, the efficacy of curcumin- and quercetin-loaded nutriosomes was comparable to their free drug solutions, with IC_50_ values for nutriosomes vs. free drug solution closely aligning (8.777 μg/mL vs. 3.949 μg/mL, and 7.020 μg/mL vs. 7.258 μg/mL, respectively) (Fulgheri et al., 2023). Nutriosomes appear to be a promising carrier system for dietary polyphenols such as curcumin and quercetin, suggesting their potential use as adjuvants in malaria treatment. However, their co-loading with artemisinin in nutriosomes has proved to be less effective, indicating a need for further optimization or alternative formulations (Fernandez-Gonzalez et al., 2021; Fulgheri et al., 2023).

##### 4.1.1.2 Surface-decorated liposomes for active targeting

In the realm of antimalarial drug delivery, biopolymers such as heparin have gained attention due to their dual functionality. Heparin not only shows potential as an antimalarial agent, but also targets infected red blood cells (pRBCs). As an antimalarial agent, heparin acts by inhibiting Plasmodium invasion of red blood cells, particularly through interactions with key proteins like the merozoite surface protein 1 (MSP1) and the circumsporozoite (CS) protein. Lantero et al. (2020) and Marques et al. (2017) have highlighted heparin’s role in disrupting the key processes of Plasmodium, such as hepatocyte invasion.

Interestingly, single-molecule force spectroscopy analysis has revealed that heparin specifically binds to pRBCs with a strength comparable to antibody-antigen interactions, highlighting its targeting potential (Marques et al., 2017).

Numerous research studies have highlighted the potential of heparin-decorated liposomes as effective delivery systems for plant-derived drug products with antimalarial properties. For example, Ledoux et al. (2020) explored the use of heparin as a ligand in nanocarrier formulations for the encapsulation of poupartone B, a phytochemical compound from *Poupartia borbonica* leaves. Due to its negative charge, heparin was electrostatically bound to cationic liposomes consisting of DOTAP, DOPC and Cholesterol (4:76:20 M ratio). Poupartone B-loaded liposomes containing heparin (1.3 µg heparin/mM liposome lipids) exhibited superior antimalarial activity against both artemisinin-resistant strain IPC 3445 and chloroquine-sensitive strain 3D7, compared to both free Poupartone B and Poupartone B encapsulated in liposomes without heparin. These studies suggest that the combination of heparin’s targeting potential with the natural efficacy of plant-derived compounds can enhance the overall effectiveness of malaria treatments.

While heparin-decorated liposomes have shown promise in antimalarial drug delivery, a significant concern has been identified; heparin that is only electrostatically bound to liposomes tends to detach during blood circulation. Since this detachment can result in a loss of the targeted delivery potential, further efforts have been made to covalently attach heparin to liposomes. By covalently binding heparin to primaquine-loaded liposomes, Marques et al. (2017) successfully developed a stable delivery system composed of DOPC:PE: Cholesterol: DOTAP, 46:30:20:4. This stability was achieved through crosslinking the amine groups present in the liposomes with the carboxyl groups of heparins. In tests using *P. falciparum* strains, the primaquine-loaded liposomes with covalently bound heparin showed a substantial increase in antimalarial activity compared to non-bound liposomes, reducing parasitemia from 52.1% to 16.0% at a concentration of 0.3 μg/mL of liposome-bound heparin. Moreover, heparin covalently bound to liposomes demonstrated a significant reduction in anticoagulant activity compared to heparin which was electrostatically bound.

The inherent anticoagulant property of heparin, while beneficial in certain medical applications, poses significant risks in others, especially where uncontrolled bleeding or haemorrhagic complications could be a concern. Despite its combined targeting and antimalarial potentials, the use of heparin in malaria treatment has been limited due to its strong anticoagulant activity, which raises the risk of intracranial bleeding. The balancing act between leveraging heparin’s drug delivery benefits and mitigating its anticoagulant effects is critical. This necessitates the development of modified heparin formulations or alternative strategies that retain the targeting potential of heparin while minimizing its anticoagulant impact (Burns et al., 2019; Ismail et al., 2019).

Recent advancements in heparin modification for antimalarial applications have shown promising results. Lantero et al. (2020) explored chemically modified heparin, including depolymerization, desulfation, oversulfation and conjugation to primaquine, using unfractionated heparin (>12 kDa) obtained from animal mucosae. These modifications aimed to retain the antimalarial activity while potentially mitigating the adverse anticoagulant effects. They reported that the 2-O-desulfation of ioduric acid (IdoA) in heparin significantly reduced its anticoagulant activity while moderately affecting its antimalarial properties. However, altering the glucosamine N-sulfate to an acetyl group in heparin led to the loss of its antimalarial activity, highlighting the delicate balance in chemical modifications.

In parallel, it was found that the covalent crosslinking of heparin onto the surface of PEGylated liposomes did not impact the antimalarial activity of heparin, suggesting the feasibility of this approach in creating multifunctional, long acting and targeting antimalarial delivery systems.

#### 4.1.2 Plant-derived surface-modified nanocapsules

The realm of nanotechnology has seen significant advancements in the surface modification of nanocapsules using various agents, aiming at enhancing drug performance both *in vitro* and *in vivo*. One key area of exploration has been modifications to improve the intravenous efficiency of antimalarial drugs, including plant-derived active pharmaceutical ingredients such as quinine. Michels et al. (2019) demonstrated how nanocapsule surface modifications can significantly impact quinine’s pharmacokinetics and overall efficacy. By altering the surface properties of nanoparticles, the plasma half-life, tissue distribution and penetration of quinine into infected RBCs improved significantly, and the survival rates of malaria mice markedly increased.

The potential of plant-derived ingredients, such as soybean lecithin, in enhancing the pharmacological profiles of antimalarial drugs has been showcased through the development of surface-decorated nanocapsules.

A notable example involves the encapsulation of halofantrine hydrochloride in nanocapsules formulated with Soy lecithin (0.75% wt/vol) as the hydrophobic surfactant, Miglyol 810 (2.5% wt/vol) as the oil phase, poly (D, L-lactide) (PLA) surface-modified with grafted PEG as the oily core. These PEGylated nanocapsules demonstrated an extended circulation time in the bloodstream (with more than sixfold increase in the area under the curve for halofantrine in plasma), leading to a more sustained decrease in parasitaemia compared to traditional capsule formulations (Mosqueira et al., 2004).

#### 4.1.3 Ligand-decorated solid lipid nanoparticles

Treating cerebral malaria effectively is challenging due to the selective permeability of the blood-brain barrier, which predominantly allows only small lipid-soluble drugs to access brain cells (Wei et al., 2022).

Quinine dihydrochloride, a key drug in the treatment of cerebral malaria, could greatly benefit from enhanced brain-targeted delivery mechanisms (Gupta et al., 2010). Solid lipid nanoparticles (SLNs) have shown promise as versatile delivery systems capable of encapsulating both lipophilic and hydrophilic drugs, potentially facilitating their transport across the blood-brain barrier. Additionally, the use of transferrin, a glycoprotein, as a molecular probe for brain targeting is gaining attention (Gan and Feng, 2010).

Transferrin receptors on various cell types, including those in the brain, facilitate the transport of substances through receptor-mediated endocytosis, a feature that can be harnessed to improve the delivery of antimalarial drugs to brain cells (Gan and Feng, 2010; Gupta et al., 2010). This combination of advanced drug delivery systems and molecular targeting strategies represents a promising Frontier in the fight against cerebral malaria, potentially overcoming the current limitations posed by the blood-brain barrier.

Gupta et al. (2010) developed a novel therapeutic approach for cerebral malaria by creating transferrin-conjugated SLNs loaded with quinine dihydrochloride. The formulation of these SLNs utilized hydrogenated soya phosphatidylcholine (HSPC), triolein, cholesterol and distearyl phosphatidylethanolamine (DSPE) in a weight-to-weight ratio of 9.58:9.58:3.16:3.16. The carboxylic group of transferrin was covalently linked to the amino group of DSPE on the surface of the drug-loaded SLNs using 1-ethyl-3-(3-dimethylaminopropyl) carbodiimide hydrochloride (EDC) as the coupling agent. The resulting Transferrin-SLNs exhibited a mean diameter and zeta potential of 126.4 nm and 15.4 mV, compared to the unmodified SLNs, which had measurements of 108.2 nm and 3.7 mV, respectively.

In the same study, *in vivo* distribution studies conducted on thirty-two albino rats revealed significant differences in the organ distribution of quinine dihydrochloride. Following intravenous administration, transferrin-SLNs showed greater uptake of the drug by the brain, with drug recovery percentages of 12.40% and 3.32% after 1 h and 24 h, respectively. In contrast, the unmodified SLNs and free drug showed lower drug recovery percentages: 2.90% and 0.72% for the unmodified SLNs, and 1.64% and 0.13% for the free drug after 1 h and 24 h, respectively. This enhanced brain uptake of transferrin-SLNs is likely due to receptor-mediated transcytosis, a process normally utilized for circulating transferrin uptake, highlighting the potential of transferrin-conjugated SLNs in cerebral malaria treatment.

In another study, Emami et al. (2018) developed a nanostructured lipid carrier (NLC) for brain delivery of artemisinin. These NLCs were formulated using a solvent evaporation method with a lipid phase comprising cholesterol, Compritol^®^, oleic acid, stearylamine, soy lecithin and artemisinin, and an aqueous phase containing various concentrations of Poloxamer 188 or Tween 80.

The optimized artemisinin-NLC, which included Compritol^®^/Tween 80/Oleic acid/stearylamine/soy lecithin/Artemisinin/dichloromethane (20 mg/0.25%/5 mg/5 mg/2 mg/2 mg/2.5 mL, respectively, were then coupled with transferrin as targeting ligand. *In vitro,* cytotoxicity against the U-87MG brain cancer cell line was also investigated. An increase in particle size and mean release time was observed for the transferrin-artemisinin-NLCs, which increased from 145.0 nm at 24.0 h to 170.0 nm, at 29.2 h, respectively. However, there was a decrease in zeta potential and entrapment efficiency from 24.3 mV to 82.3% to 14.1 mV and 68.8%, respectively.

Notably, the NLCs loaded with artemisinin coupled with transferrin showed much better cytotoxicity activity against the U-87MG brain cancer cell line than non-coupled NLCs, the non-decorated NLCs and free drug. These results suggest that transferrin-artemisinin-NLCs can be used as an effective delivery system for artemisinin in treating brain tumours and malaria.

### 4.2 Surface-decorated plant virus for antimalarial drug delivery

Cowpea mosaic virus (CPMV), a non-enveloped icosahedral plant virus with an approximate diameter of 30 nm, has been characterized by near-atomic resolution (Mao et al., 2021). Its composition, consisting of 60 copies of two different types of coat protein, provides multiple attachment sites for the conjugation of various moieties on its external surface, facilitating the development of diverse functionalized nanoparticles.

In a study by Al-Refai’a (2019), Chloroquine derivatives, specifically 4-amino chloroquinoline derivatives such as 3-((7 chloroquinolin-4-yl) amino) propionic acid (CQp) and (7-chloroquinolin-4-yl) alanine chloroquine diphosphate (CQ-ala), were investigated for their antimalarial properties, both independently and when conjugated to CPMV and Ferrocene (Fc). Owing to their covalent attachment to the external surface carboxylates of the viral nanoparticle, these derivatives demonstrated beta-haematin inhibitory activity against *P. falciparum* strains 3D7 (CQ-sensitive) and Dd2 (CQ-resistant). Notably, Fc, which lacks antimalarial activity on its own, showed increased activity when conjugated with CPMV, highlighting the potential of nanoparticle conjugation in enhancing drug efficacy, the study also explored the inhibition of haem-crystallization. When compared to CQ control (58% inhibition), CQ-derivatives (CQp and CQ-ala) exhibited higher inhibition percentages (65.5% and 74.22%, respectively), and their conjugation with Fc further improved the inhibition percentages to 70.82% and 89.89%, respectively.

*In vitro* activities of these conjugated compounds against *P. falciparum* 3D7 (CQ sensitive) and Dd2 (CQ resistant) strains showed that the CPMV-CQ-ala-Fc conjugate exhibited superior activity, with IC_50_ values of 0.2087 nM and 0.1352 nM, respectively. The acidic environment (pH 4.5) of the *P. falciparum*’s food vacuole, which is targeted by the CPMV conjugates, facilitates the degradation of nanomaterials and subsequent drug release. CPMV nanoparticles, serving as carrier vehicles, provide a smart mechanism for delivering 174 Fc molecules through the membranes of infected erythrocytes, ensuring a higher dose reaches the target and effectively destroys resistant parasites. Together, these findings demonstrate great the potential of CPMV functionalization for the targeted delivery of antimalarial drugs.

### 4.3 Surface-decorated mesoporous silica nanocarriers

**FIGURE 5:**
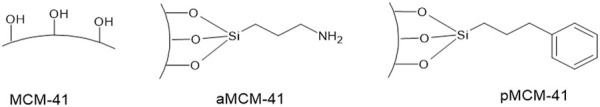
Schematic illustration of surface functional groups of mobile crystalline material 41 (MCM-41), 3-aminopropyl silane (aMCM-41), and 3-phenylpropyl silane (pMCM-41) cyclodextrin-based nanoformulations. Structures created with ChemSketch 2021.2.1 version C35E41.

Recent studies have focused on the encapsulation and release profiles of quinine using three silica nanocarriers functionalized with mobile crystalline material 41 (MCM-41), 3-aminopropyl silane (aMCM-41) and 3-phenylpropyl silane (pMCM-41), as shown in Figure 5 (Hirayama et al., 2021). Of all these functionalized nanoparticles, pMCM-41 demonstrated superior quinine-loading capacity, entrapment and release profile compared to MCM-41 and aMCM-41. The drug-loading capacity and entrapment efficiency for pMCM-41 were 7.48% and 49.72%, respectively (*versus* 2.73% and 12.50% for MCM-41, and 1.06% and 2.74% for aMCM-41). The excellent performance for these pMCM-41 nanocarriers was attributed to enhanced π-π interactions between the delocalized π electrons of the 3-phenylpropyl silane and quinine, improving drug loading and release performance. Furthermore, p-MCM 41 exhibited a controlled release of quinine at 25°C and pH 7, positioning it as an ideal bio-nanomaterial for quinine delivery.

Anti-plasmodial activity assessments were done using various formulation compositions: MCM-41 loaded with quinine or artemisinin, pMCM-41 loaded with quinine, and 3-aminopropyl silane functionalized loaded with artemisinin. Data indicated that MCM-41 loaded with quinine had the highest antimalarial activity, with ED50 values of <0.062 mg/kg body weight after 4 days of inoculation, remaining constant after 8 days. Quinine-loaded MCM-41 was followed by MCM-41 loaded with artemisinin (ED50 0.113 mg/kg), aMCM-41 loaded with artemisinin (ED50 0.327 mg/kg), and pMCM-41 loaded with quinine (ED50 0.157 mg/kg). This suggests that MCM-41 is a promising system for sustained drug release and efficient adsorption by infected red blood cells (Amolegbe et al., 2018).

## 5 Challenges associated with plant-based nanotechnologies

Plant-based NPs face significant challenges and limitations in their development pipeline for malaria management. These include achieving consistency and stability in NPs preparations, and ensuring treatment efficacy, safety, and accessibility, as well as regulatory approval. Addressing these challenges is vital for transitioning plant-based NPs from laboratory research to practical, clinical applications, particularly in the management of malaria in low-income regions. The following paragraphs provide a brief highlight to illustrate the challenges associated with plant-based nanoparticles in different contexts.

***Protocol standardisation***—Looking at the tabulated data summarized in the previous sections, there are evident discrepancies in experimental conditions used to investigate plant-based nanoparticles. These evolve from the variability in NPs characteristics (e.g., particle size) to generic experimental conditions such as NPs concentrations (i.e., LC_50_), which have been expressed in ppm or in weight by volume, without considering the number of NPs that are effectively involved in the biological media. Other varying parameters such as cell types, culture media, animal models, and procedures (Luzala et al., 2023), may also hamper the comparability of nanomedicine efficacy studies. Standardizing these protocols is essential for advancing clinical research (Najer et al., 2014; Sidhaye et al., 2016; Boateng-Marfo et al., 2018; Mukubwa et al., 2020; Shater et al., 2023).

***Targeting and delivery***—Enhancing the targeted delivery of antimalarials through NPs surface decoration is crucial for addressing the challenges posed by the variability in pharmacokinetic profiles, efficacy and safety of various drugs, but also particularly the infections by *Plasmodium vivax* and *Plasmodium ovale*, which form dormant hypnozoites in the liver and contribute to drug resistance (Flannery et al., 2022; Wang et al., 2023). Current research has developed various strategies for NP surface decoration to improve drug targeting precision (Nisini et al., 2018). However, consistently controlling the ligand density and configuration on NPs surfaces poses significant challenges, impacting both preclinical efficacy and safety. This variability is eventually set to complicate product scalability and regulatory approval, as production processes must be adjusted for industrial scale-up. Standardizing the surface decoration methods is therefore critical for transitioning NPs from experimental treatments to widely available therapies, particularly in malaria-affected regions.

***Safety and Toxicity—*** Environmental impacts and toxicity profiles are critical concerns. Plant-based metallic and metal oxide NPs may accumulate in ecosystems due to inadequate environmental management in low-income countries. This accumulation can lead to contamination of water bodies, affecting aquatic life and communities relying on untreated river water (Benelli et al., 2018). Additionally, the skin permeability of essential oil-based nanoformulations, particularly in children under two, necessitates further investigation into their safety and biocompatibility (Manion and Widder, 2017). Some NPs also exhibit intrinsic antimalarial activity without the need for activation, but their long-term safety remains unproven (Zhang et al., 2020). The development of antimalarial vaccines using plant viruses is another demanding area that requires meticulous developments to yield safer delivery mechanisms as well as acceptable adjuvants, such as Abisco-100 (as discussed earlier in section 3.1.3). For instance, the hemolytic potential of saponin-based adjuvants has limited further vaccines development (Huis in ’t Veld et al., 2022; Soltani et al., 2014; Vo et al., 2017; Wang et al., 2007), necessitating careful molecular engineering to prevent exacerbating malaria-induced anemia.

***Manufacturing and scalability***—Reproducibility and standardization are significant obstacles in scaling up plant-based NPs for malaria treatment. The variability in the quantity and quality of phytochemicals (Kunle et al., 2012) requires the standardization of the harvesting conditions of the plant materials to ensure global consistency in production. While these nanoformulations are cost-effective at the laboratory bench, scaling to industrial manufacturing encounters economic challenges, potentially limiting accessibility in low-income countries. Key factors such as long-term stability, zeta potential, size homogeneity, and optimal storage conditions must be addressed to prevent NPs degradation and ensure consistent performance. Additionally, parameters like surface characteristics, dosage, encapsulation efficiency, loading capacity, release profiles, effective dose, and biodistribution need optimization for successful scale-up (Hua et al., 2018; Jin et al., 2022). Addressing these challenges is crucial for transitioning plant-based NPs from lab research to industrial applications following good practices manufacturing (GMP) guidelines and enabling their clinical use in managing malaria in resource-limited settings.

***Regulatory Challenges***—The regulatory landscape for nanotechnologies, particularly plant-based nanoparticles in malaria management, is complex and still evolving. Oversight agencies face the challenge of defining clear guidelines that balance innovation with safety and environmental protection. The unique properties of nanoparticles, such as increased reactivity and potential bioaccumulation, necessitate rigorous assessment protocols to evaluate their toxicity, efficacy, and environmental impact. Currently, there is a lack of standardized methodologies for testing and validating the safety of nanomaterials, which complicates regulatory approvals. Additionally, discrepancies between different countries’ regulatory frameworks can hinder international collaboration and the global distribution of nanotechnology-based treatments. For successful clinical and commercial deployment of plant-based nanoparticles, establishing harmonized regulatory standards and frameworks is crucial. This would facilitate smoother development pathways and help ensure that these innovative treatments are both safe and accessible to those in need, especially in regions burdened by malaria.

## 6 Importance of interdisciplinary collaboration

**FIGURE 6:**
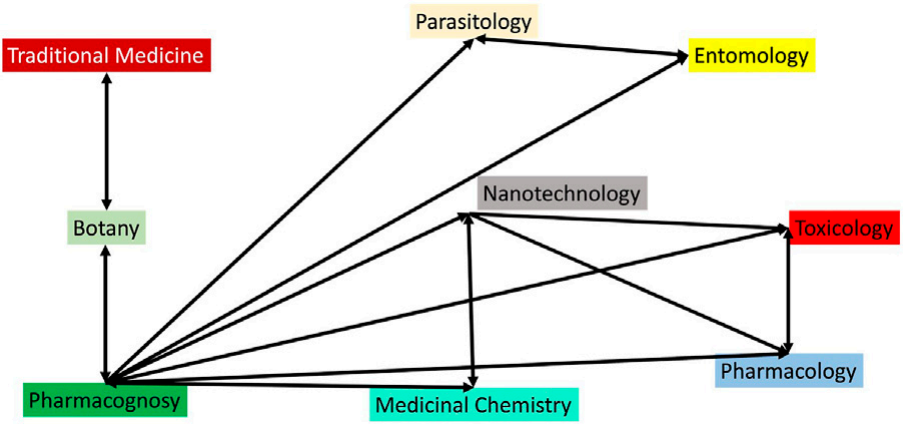
Description of the interdisciplinarity connections between various disciplines enabling plant-based NPs development from the identification of plant materials to their application in nanotechnology for malaria management.

Interdisciplinary collaborations are crucial for advancing plant-derived nanotechnology in malaria management. The integration of fields such as parasitology, toxicology, ecological biosafety, intracellular trafficking, biomaterials, polymer science, molecular and cellular immunology, and physico-chemistry is essential to develop effective prevention and treatment tools (Mohammadi et al., 2021). This section briefly discusses how a wide range of disciplines—including traditional medicine, botany, pharmacognosy, entomology, biology, medicinal chemistry, nanotechnology, pharmacology, and toxicology—contribute to the development of plant-based nanoformulations for larvicidal activities, antimalarial effects, or vaccine production aimed at combating malaria (Figure 6).

***Traditional medicine*** *—*Approximately 80% of the global population relies on plant-based traditional healthcare products for their primary healthcare needs (Noronha et al., 2020). A diverse range of indigenous plants are utilized in the prevention, treatment, and management of malaria across various cultures (Botsaris, 2007; Kweka et al., 2008; Mavundza et al., 2013; Habibi et al., 2022; Nigussie and Wale, 2022). Countries like Brazil, Nigeria, Ethiopia, Kenya, Ghana, and Cameroon have documented plant species traditionally employed to combat malaria and fever, highlighting the rich botanical knowledge that underpins traditional practices (Olorunniyi and Morenikeji, 2013; Iyamah and Idu, 2015; Alebie et al., 2017; Milliken et al., 2021). Historical studies have shown a significant interest in indigenous plants for pharmaceutical applications, particularly since major antimalarial drugs such as quinine and artemisinin originated from traditional medicinal plants (Graz et al., 2011; Alebie et al., 2017). This underlines the potential of traditional medicine as a resource for developing new therapeutic agents in the form of plant-based nanoparticles aimed at improving malaria outcomes."

***Botany*** *—*The vast diversity of plant species, along with climate change-related shifts in their geographic distributions, underscores the importance of accurate plant identification upon discovery through traditional medicine (Cope et al., 2012; Alaerts et al., 2014). The genus *Artemisia L.* of the family *Asteraceae*, for example, presents significant taxonomic challenges due to its complexity (Sancar et al., 2021). *Artemisia annua*, the source of the antimalarial compound artemisinin, is frequently misidentified and can be adulterated or substituted with morphologically similar species within the genus, such as *Artemisia argyi, Artemisia absinthium, Artemisia leucophylla*, and *Artemisia lavandulaefolia* (Nazar and Mahmood, 2011; Song et al., 2016; Liu et al., 2022). To ensure the authenticity and purity of this medicinal plant, sophisticated identification techniques such as phytochemical screening, DNA sequencing, or microscopic analyses are essential (Nazar and Mahmood, 2011; Lan et al., 2022).

***Pharmacognosy*** *—*The urgent need for standardization and authentication of medicinal plant materials used in traditional malaria remedies is critical. Establishing standardization parameters such as organoleptic characteristics, macroscopic observations (morphological descriptions), microscopic evaluations (anatomical studies), and physicochemical analyses of plant material is essential. These parameters help verify the identity and purity of crude drugs derived from wild sources, ensuring quality control (Chanda, 2014; Ogunkunle et al., 2014; Ogunkunle et al., 2021). Furthermore, scientific validation studies on the antimalarial, larvicidal, and mosquito repellent activities of traditional medicinal plants like *Laggera pterodonta, Brucea javanica,* and *Samanea saman* are vital. Such ethnopharmacological research aims to confirm their preventive or curative effects, ensuring the reproducibility and efficacy of these natural products (Okhale et al., 2010; Namsa et al., 2011; Chanda, 2014; Thazar et al., 2017). These efforts are crucial for integrating traditional remedies into mainstream healthcare, particularly in regions heavily affected by malaria.

***Parasitology*** *—*Understanding host-parasite interactions during infection is crucial in the preclinical development phase of antimalarial drugs and vaccines (Acharya et al., 2017; Burgert et al., 2020). Preclinical studies typically use murine models and experimental human malaria infections to assess the efficacy of potential antimalarial drugs. These studies help to determine how effectively a drug can control or eliminate malaria parasites within the host. For vaccine development, it is essential to understand the the immune envasion strategies employed by *Plasmodium* (Hafalla et al., 2011). Researchers focus on stage-specific proteins of *Plasmodium*, which are critical targets for the host’s immune response (Chandley et al., 2023). One such target is the P25 protein, expressed in the parasite’s gametes, zygote, and ookinete stages, and recognized as a primary focus for vaccine strategies (Carter et al., 2000). These insights into the parasite’s lifecycle and immune evasion mechanisms are vital for identifying drug targets, and designing vaccines that can effectively stimulate the host’s immune system to combat malaria.

***Entomology*** *—*Vector control is a pivotal aspect of disease management, aiming primarily at vector elimination (Shaukat et al., 2010; Animut and Lindtjørn, 2018; Subbarao et al., 2019). Entomological surveys play a critical role in malaria vector control programs, guiding operational activities and research efforts to track and manage mosquito populations effectively (WHO, 2013). In the face of rising resistance to conventional insecticides such as DDT and permethrin, molecular entomology has been instrumental in identifying resistant Anopheles mosquito species (Collins et al., 2000; Oduola et al., 2012). This growing resistance has prompted the exploration of plant-based extracts and nanoparticles for vector control, as resistance to these natural substances is relatively rare (Burfield and Reekie, 2005). Additionally, essential oils, traditionally used to deter mosquito bites, are being investigated as viable alternatives to synthetic insecticides, offering a potentially effective and sustainable approach to vector management (Bossou et al., 2013).

***Medicinal chemistry*** *—*Medicinal chemistry plays a critical role in the exploration of traditional medicinal plants for malaria prevention and treatment. Numerous studies have been conducted to perform qualitative and quantitative analyses of the major bioactive constituents of these plants (Medhi et al., 2010; Bankole et al., 2016). Various active compounds have been isolated from plants known in traditional medicine for their antimalarial properties (Bero et al., 2009; Kayembe et al., 2010). Through bioassay-guided fractionation, several promising molecules have been identified, such as demethoxymatteucinol from *Bauhinia purpurea* L., 12,16-dideoxy aegyptinone B from *Zhumeria majdae*, and 1-isopropyl-4-methylbicyclo [3.1.0]hexan-3-one from *Elaeagnus indica*. These compounds have shown notable antimalarial or larvicidal effects, highlighting the potential of traditional plants as sources for novel therapeutic agents (Boonphong et al., 2007; Moein et al., 2008; Srinivasan et al., 2015).

***Nanotechnology*** *—*In response to the emergence of multidrug-resistant malaria strains, nanomaterials have become a promising solution to address several limitations associated with emerging natural drug products, traditional herbal medicines used in malaria treatment and vector control. These challenges include *in vivo* instability, low bioavailability, poor absorption, low solubility, and insufficient target specificity, which are often encountered with bulk materials (Benelli and Govindarajan, 2017; Mohammadi et al., 2021; AlGabbani, 2023). To enhance efficacy and target delivery, various nanostructured formulations such as metal nanoparticles (MeNPs), liposomes, nanoemulsions, nanogels, polymeric nanoparticles, micelles, and solid lipid nanoparticles have been developed using plant extracts or isolated compounds (Veerakumar et al., 2013; 2014; Govindarajan and Benelli, 2016a). These nanoformulations have shown improved efficiency and pharmacokinetics compared to their bulk material counterparts, demonstrating significant potential of plant-based nanotechnologies in the management of malaria (Benelli and Govindarajan, 2017; Chaves et al., 2022; Dubey and Mostafavi, 2023).

***Pharmacology*** *—*The incorporating of bioactive compounds into nanoparticles derived from plants has proven to enhance the physicochemical and pharmacokinetic/pharmacodynamic properties, specificity, efficacy, and safety of pharmaceutical agents (Onoue et al., 2014; Abdifetah and Na-Bangchang, 2019; Le et al., 2019). Plant-derived nanocarriers have significantly improved the drug substances that have been used as mosquito repellents, larvicides, and antimalarials. Comprehensive studies have assessed various aspects of these nanoformulations, including stability, entrapment efficiency, drug release profiles, targeted delivery, efficacy, bioavailability, and toxicity profiles (Le et al., 2019; Manconi et al., 2019; Ledoux et al., 2020; Kamaraj et al., 2023). These advancements highlight how pharmacological studies enable the potential of plant-derived nanoparticles to revolutionize the delivery and effectiveness of malaria management strategies.

***Toxicology*** *—*Evaluating the biosafety of nanomaterials on non-targeted organisms is crucial, particularly for the widespread application and commercialization of these materials. The biosafety assessments include a range of non-target organisms, such as fish species (*Gambusia affinis*, *Zebra danio*, and *Poecilia reticulata*), dragonfly nymphs (*Diplonychus annulatum*), and Green monkey kidney cells, which help in determining the ecological impact of nanoproducts (Patil et al., 2012; Kamaraj et al., 2023; Shater et al., 2023; Shamseldean et al., 2024). To enable large-scale application and commercialization, it is essential to ensure that these nanomaterials target the intended species effectively while minimizing any adverse effects on non-target organisms (Mandodan et al., 2023). For example, AgNPs derived from *Solanum xanthocarpum* have demonstrated significant larvicidal effects against *Anopheles stephensi l*arvae, without harming the non-target organism model, *P. reticulata*, which shows their potential as eco-friendly solutions for mosquito vector control (Kumar et al., 2022). This balance is key to the responsible development and deployment of nanotechnologies in environmental settings.

## 7 Conclusion

Malaria remains a leading cause of morbidity and mortality in low-income countries globally, with infection dynamics involving the *Plasmodium* spp. parasites, particularly *P. falciparum*, the most virulent species. Transmission is facilitated by the female Anopheles mosquitoes, notably *Anopheles stephensi* and *Anopheles gambiae*, with humans serving as the intermediate host, where the parasite matures and becomes infectious. To combat malaria, efforts have been directed both upstream, through vector control strategies aimed at interrupting the mosquito life cycle of the parasites, and downstream, via the administration of antimalarial drugs to halt the parasite’s development within the host. However, the efficacy of traditional antimalarial agents, such as DDT, pyrethroids, chloroquine and even the newer artemisinin derivatives, has been compromised over time due to the development of resistance by both vectors and parasites. Additionally, the environmental and human toxicity of these agents poses further challenges.

This review highlighted the evolving landscape of antimalarial strategies, noting the potential of plant-based nanotechnologies in overcoming the above-mentioned challenges in malaria treatment and prevention. The exploration of plant-based nanotechnologies presents a groundbreaking approach to combating malaria, particularly in developing countries where the disease remains a prominent health challenge. By harnessing the unique properties of nanoparticles, including enhanced solubility, stability and targeted delivery potential, these technologies offer a promising avenue for overcoming the limitations of traditional antimalarial treatments and vector control methods. Nanoparticle formulations derived from plant materials not only exhibit reduced toxicity but also counteract the growing resistance against conventional antimalarial drugs and pesticides. Functionalizing such nanoparticles to specifically target malaria offers promising avenues for improving drug pharmacokinetics, reducing toxicity, and enhancing patient adherence to treatment. The targeted delivery of antimalarial drugs through nanoparticles facilitates direct action on the parasites within infected erythrocytes and the Anopheles mosquitoes, thereby ensuring a more effective and efficient treatment regimen. Furthermore, nanoparticles intrinsically active against the Anopheles mosquitoes introduce a novel vector control strategy that could significantly reduce transmission rates.

Collectively, plant-based nanotechnologies embody a holistic strategy that addresses the multifaceted challenges of malaria eradication in developing countries, from enhancing drug efficacy and patient adherence to innovating vector control and preventive measures. This approach not only signifies a substantial leap forward in malaria treatment but also highlights the potential of nanotechnology in revolutionizing global health initiatives against infectious diseases.
